# VER/VEGF receptors regulate AMPA receptor surface levels and glutamatergic behavior

**DOI:** 10.1371/journal.pgen.1009375

**Published:** 2021-02-09

**Authors:** Eric S. Luth, Molly Hodul, Bethany J. Rennich, Carmino Riccio, Julia Hofer, Kaitlin Markoja, Peter Juo

**Affiliations:** 1 Department of Developmental, Molecular, and Chemical Biology, Tufts University School of Medicine, Boston, Massachusetts, United States of America; 2 Department of Biology, Simmons University, Boston, Massachusetts, United States of America; 3 Program in Neuroscience, Graduate School of Biomedical Sciences, Tufts University School of Medicine, Boston, Massachusetts, United States of America; Brown University, UNITED STATES

## Abstract

Several intracellular trafficking pathways contribute to the regulation of AMPA receptor (AMPAR) levels at synapses and the control of synaptic strength. While much has been learned about these intracellular trafficking pathways, a major challenge is to understand how extracellular factors, such as growth factors, neuropeptides and hormones, impinge on specific AMPAR trafficking pathways to alter synaptic function and behavior. Here, we identify the secreted ligand PVF-1 and its cognate VEGF receptor homologs, VER-1 and VER-4, as regulators of glutamate signaling in *C*. *elegans*. Loss of function mutations in *ver-1*, *ver-4*, or *pvf-1*, result in decreased cell surface levels of the AMPAR GLR-1 and defects in glutamatergic behavior. Rescue experiments indicate that PVF-1 is expressed and released from muscle, whereas the VERs function in GLR-1-expressing neurons to regulate surface levels of GLR-1 and glutamatergic behavior. Additionally, *ver-4* is unable to rescue glutamatergic behavior in the absence of *pvf-1*, suggesting that VER function requires endogenous PVF-1. Inducible expression of a *pvf-1* rescuing transgene suggests that PVF-1 can function in the mature nervous system to regulate GLR-1 signaling. Genetic double mutant analysis suggests that the VERs act together with the VPS-35/retromer recycling complex to promote cell surface levels of GLR-1. Our data support a genetic model whereby PVF-1/VER signaling acts with retromer to promote recycling and cell surface levels of GLR-1 to control behavior.

## Author summary

Sensation, behavior, and cognition all depend on the proper function of neuronal connections called synapses. Synapses that use the neurotransmitter glutamate to signal between nerve cells are the most abundant type in our brain. Presynaptic neurons release glutamate, which activates glutamate receptors on postsynaptic neurons. Dysfunction of glutamate synapses leads to several neurological disorders, and changing their strength–in part by altering glutamate receptors numbers on the surface of the postsynaptic cell—provides the cellular basis of learning and memory. Much remains to be learned about how factors released from other cell types affects synaptic communication. We took advantage of light-activated molecular switches engineered into specific sensory neurons of *C*. *elegans* worms to trigger a behavioral reflex that depends on glutamate synapses. Using this behavior, we identified proteins called VER-1 and VER-4 as important for glutamate synapse function. We found that worms missing these VER proteins or their activator PVF-1 have reduced levels of glutamate receptors at the postsynaptic surface and defects in glutamate-dependent behaviors. Our results suggest that inter-tissue cross-talk between muscle PVF-1 and neuronal VERs is important for controlling the number of glutamate receptors at the cell surface, robust neuronal communication and behavioral responses.

## Introduction

Regulation of the number of AMPA receptors (AMPARs) at synapses alters synaptic strength and is a major mechanism underlying learning and memory [1]. Dysregulation of the development or function of AMPAR-containing synapses is linked to the pathogenesis of several neurological disorders including autism spectrum disorders [2]. Thus, understanding the molecular mechanisms that control AMPAR abundance at synapses is critical to understanding physiological and pathological alterations in synaptic signaling.

Synaptic AMPAR levels are regulated by several trafficking pathways including motor-dependent transport from the soma, membrane insertion, lateral diffusion and endocytosis [3]. Internalized AMPARs are either targeted for lysosomal degradation or reinserted into the plasma membrane through recycling pathways critical for synaptic function and plasticity [4], such as those mediated by recycling endosome proteins Syntaxin 13, Rab 11 and GRASP1 [5–7], or the VPS35 retromer recycling complex [8–11]. While much has been learned about these intracellular trafficking pathways, a major challenge is to understand how extracellular factors, such as growth factors, neuropeptides and hormones, regulate select AMPAR trafficking pathways to alter synaptic signaling and behavior. Previous studies identified several neuronal or astrocyte secreted factors that regulate AMPAR function or plasticity, including Narp [12], TNF-α [13], glypicans [14], and netrin-1 [15], however, the role of many other extracellular factors that regulate glutamatergic synapses remain poorly understood.

Here we identify vascular endothelial growth factor (VEGF) receptor (VEGFR) homologs *ver-1* and *ver-4* in a behavioral screen in *C*. *elegans* for genes that regulate glutamate signaling. VEGF signaling in mammals is essential; knock-out of any of the three VEGFR family members in mice results in embryonic lethality due to vascular defects [16]. VEGF also functions outside the cardiovascular system and is an important regulator of neuronal processes including neurogenesis [17,18], dendrite development and synaptic plasticity [19–21]. While much is known about how VEGFRs control vascular development, the mechanisms by which neuronal VEGFRs signal to control synapse function and behavior remain largely unexplored. A recent study showed that co-administration of exogenous VEGF and NMDA can increase AMPAR surface levels and excitatory transmission in cultured hippocampal neurons [21]. However, how endogenous VEGF and its neuronal VEGFRs regulate specific AMPAR trafficking pathways to control behavior has not been investigated.

The *C*. *elegans* VER family of receptor tyrosine kinases and their putative ligand PVF-1/VEGF are structurally and functionally conserved with their mammalian counterparts. VERs are single-pass transmembrane proteins with 6–7 extracellular Ig domains, that are ~30–50% similar to human VEGFR Ig domains, and an intracellular split tyrosine kinase domain that is ~60% similar to that of human VEGFRs [22]. In *C*. *elegans*, *pvf-1* or *ver* mutants have no obvious gross developmental defects on their own but enhance male tail defects in semaphorin/plexin mutants [23]. Expression of mammalian VEGF or VEGFRs can rescue the corresponding worm mutant defects [23], suggesting that VEGF signaling appears to be functionally conserved between *C*. *elegans* and mammals. Additionally, *C*. *elegans* PVF-1 binds and activates vertebrate VEGFRs to promote tube formation in endothelial cultures and angiogenesis in chick embryos [24]. VERs can also act in glia; v*er-1* is upregulated in *C*. *elegans* AMsh glia where it is required for glial remodeling during the dauer developmental stage [25].

Here we show that VEGF signaling regulates glutamatergic behavior and promotes cell surface levels of GLR-1 AMPARs in *C*. *elegans* neurons. Our data suggest that PVF-1/VER signaling regulates GLR-1 surface levels by acting in the same genetic pathway as VPS-35/retromer to promote recycling.

## Results

### *ver-1* and *ver-4* mutants have a specific defect in glutamate signaling

We identified *ver-1* and *ver-4*, homologs of mammalian VEGFRs, in a *C*. *elegans* RNAi screen for genes that regulate glutamatergic behavior. Gentle touch to the nose of the worm activates a glutamatergic, mechanosensory reflex called the nose touch response [26–29]. Nose touch is detected by several neurons in the head including the glutamatergic sensory neuron pair ASH [27,30–32]. ASH is a polymodal sensory neuron activated by multiple stimuli including volatile repellants, high osmolarity and light touch [27,33,34]. Light touch stimulation of ASH activates GLR-1 AMPA receptors on command interneurons including AVA [26,28,35,36] resulting in subsequent activation of motor neurons and backward locomotion [37]. Expression of channelrhodopsin (ChR2) specifically in ASH [38] enabled us to photostimulate a population of worms after RNAi-mediated gene knockdown and monitor the locomotor reversal response (optoASH assay)(S1A Fig). Optogenetic activation of ASH has previously been shown to lead to glutamate-dependent Ca^2+^ transients in and depolarization of interneurons including AVA [39,40].

**Fig 1:**
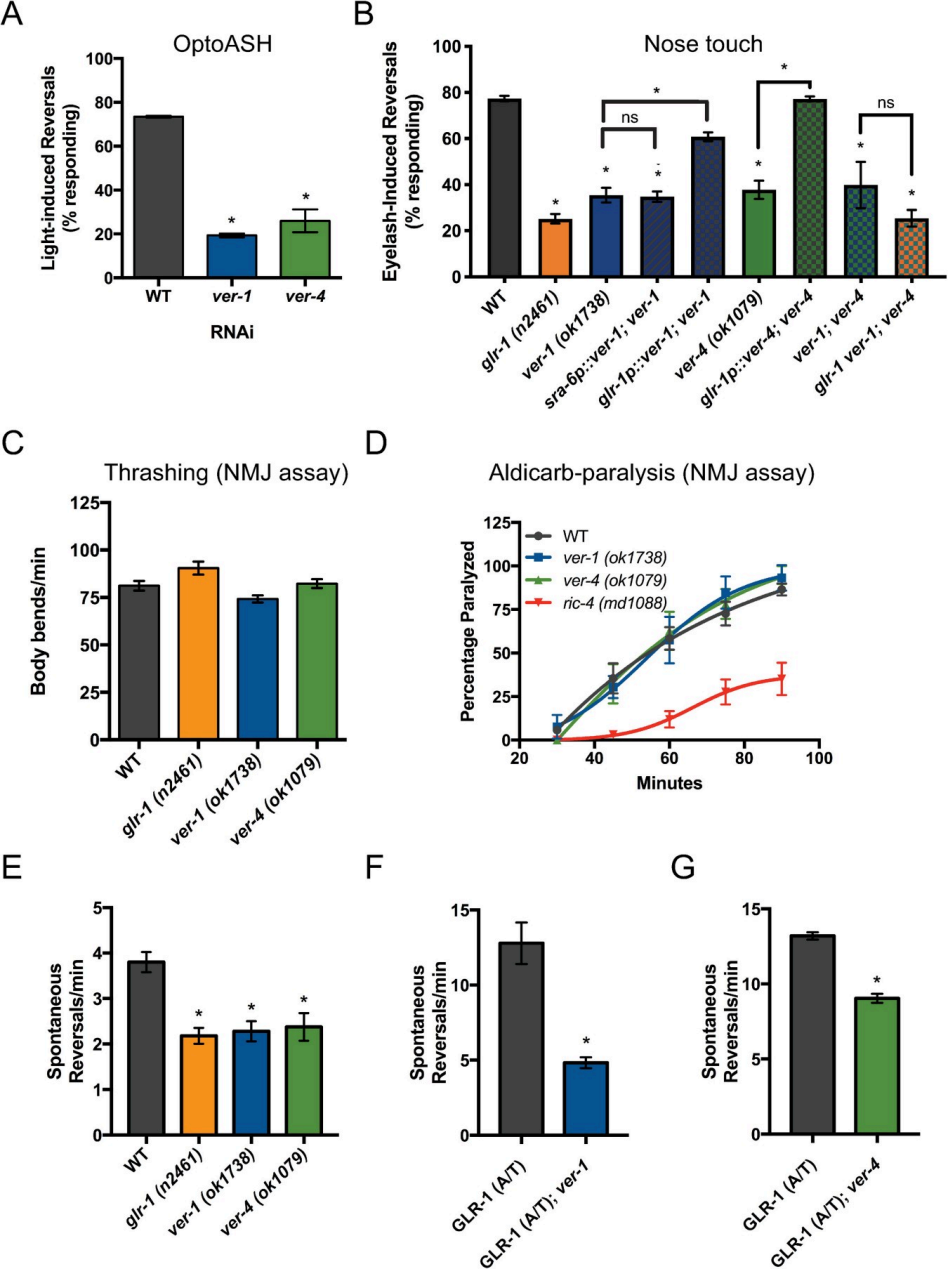
VER-1 and VER-4 act in GLR-1-expressing neurons to promote glutamate-dependent behaviors. (A) Responses to optogenetic activation of ASH after RNAi. (B) Responses to nose touch for the indicated mutant and transgenic rescue strains are shown. Rates of thrashing (C) and aldicarb-induced paralysis (D) are shown. *ric-4*/SNAP25 mutants are shown as a positive control. Spontaneous locomotion reversal frequencies in the absence (E) or presence of constitutively-active GLR-1 (A/T)(F, G). Mean ± SEM are shown (n ≥ 24 worms from ≥ 3 experiments). Values that differ significantly from wild type are indicated by asterisks above each bar, whereas other comparisons are marked by brackets. *p ≤ 0.05, ANOVA followed by Dunnett’s multiple comparison test (for A, E, F and G) or Tukey’s multiple comparison test (for B).

Control experiments showed that worms with mutations in known genetic regulators of the nose touch response including *glr-1* [26,28], the presynaptic vesicular glutamate transporter (*VGLUT) eat-4* [29], and a deubiquitinating enzyme *usp-46* that controls synaptic levels of GLR-1 [41], exhibit defects in the optoASH assay as expected (S1B and S2 Figs) [39]. We performed an RNAi screen of genes with cell adhesion molecule domains in a strain with enhanced neuronal sensitivity to RNAi [42](see Materials and Methods). Several candidates that showed strong optoASH defects after RNAi knockdown were identified including *ver-1* and *ver-4* (Figs 1A and S3). To test whether *ver-1* and *ver-4* had roles in a more physiological behavior, we tested *ver-1* and *ver-4* loss of function mutants in the traditional mechanosensory nose touch assay. We found that *ver-1* and *ver-4* mutants exhibited defects in the nose touch assay that were not significantly different from *glr-1* mutants (n.s., p = 0.29 vs *ver-1* and p = 0.16 vs *ver-4)* (Figs 1B and S3). The *ver-1 (ok1738)* allele contains an in-frame deletion that removes part of the extracellular region (amino acids 168–417) including Ig domains 2 and 3, which based on homology to mammalian VEGFRs are predicted to bind ligand [43,44]. The *ver-4 (ok1079)* allele is comprised of a ~1kb deletion in the middle of the extracellular domain that is predicted to delete most of Ig domains 4–6, which are required for VEGFR dimerization and activation [45]. This deletion results in a frameshift and premature stop codon that would encode a truncated protein lacking both the transmembrane and intracellular kinase domain. Both *ver-1 (ok1738)* and *ver-4 (ok1079)* alleles were predicted to be putative null alleles by Dalpe et al. [23] as they encode for receptors that are either unable to bind ligand or unable to insert in the membrane or dimerize and activate, and thus represent functional nulls for receptor signaling. We also found that *ver-1;ver-4* double mutants have reduced nose touch responses that are identical to *ver-1* (n.s., p = 0.99) or *ver-4* (n.s., p = 0.99) single mutants (Fig 1B). This genetic data suggests that VER-1 and VER-4 act together in the same pathway, perhaps as a heteromer as has been shown for vertebrate VEGFRs [16,46], to regulate glutamatergic behavior. Furthermore, we found that *glr-1 ver-1;ver-4* triple mutants have decreased nose touch responses that are not significantly different from each respective single mutant (n.s., p = 0.99 vs *glr-1*, p = 0.68 vs *ver-1* and p = 0.42 vs *ver-4*) or *ver-1;ver-4* double mutants (n.s., p = 0.51) (Fig 1B), suggesting that *ver-1* and *ver-4* act to regulate glutamatergic behavior in the same genetic pathway as *glr-1*.

Importantly, we found that *ver-1* and *ver-4* mutants have no obvious defects in neuromuscular junction (NMJ) function based on the rate of thrashing (Figs 1C and S3) or muscle paralysis induced by the acetylcholinesterase inhibitor aldicarb (Fig 1D and S1 Table)[47,48]. Since worm NMJ function is mediated by a balance of acetylcholine and GABA signaling, these data show that *ver-1* and *ver-4* mutants do not have a general defect in synaptic transmission and are consistent with a specific defect in glutamate signaling.

### VER-1 and VER-4 act in GLR-1-expressing neurons to promote glutamatergic behavior

We performed cell-specific rescue experiments to determine in which cells *ver-1* and *ver-4* function to regulate glutamatergic behavior. Because *ver-1* loss of function results in defects in the optoASH and the nose touch assays, we tested if *ver-1* might function in ASH. Expression of wild-type *ver-1* cDNA in ASH using the *sra-6* promoter (*sra-6p*, which also expresses in ASI and PVQ) did not rescue the *ver-1* mutant nose touch defect (Fig 1B). In contrast, expression of *ver-1* or *ver-4* cDNA in GLR-1-expressing interneurons (using *glr-1p*) rescued their respective mutant nose touch defects (Fig 1B). These data suggest that VER-1 and VER-4 can act in GLR-1-expressing neurons to promote glutamatergic behavior.

**Fig 2:**
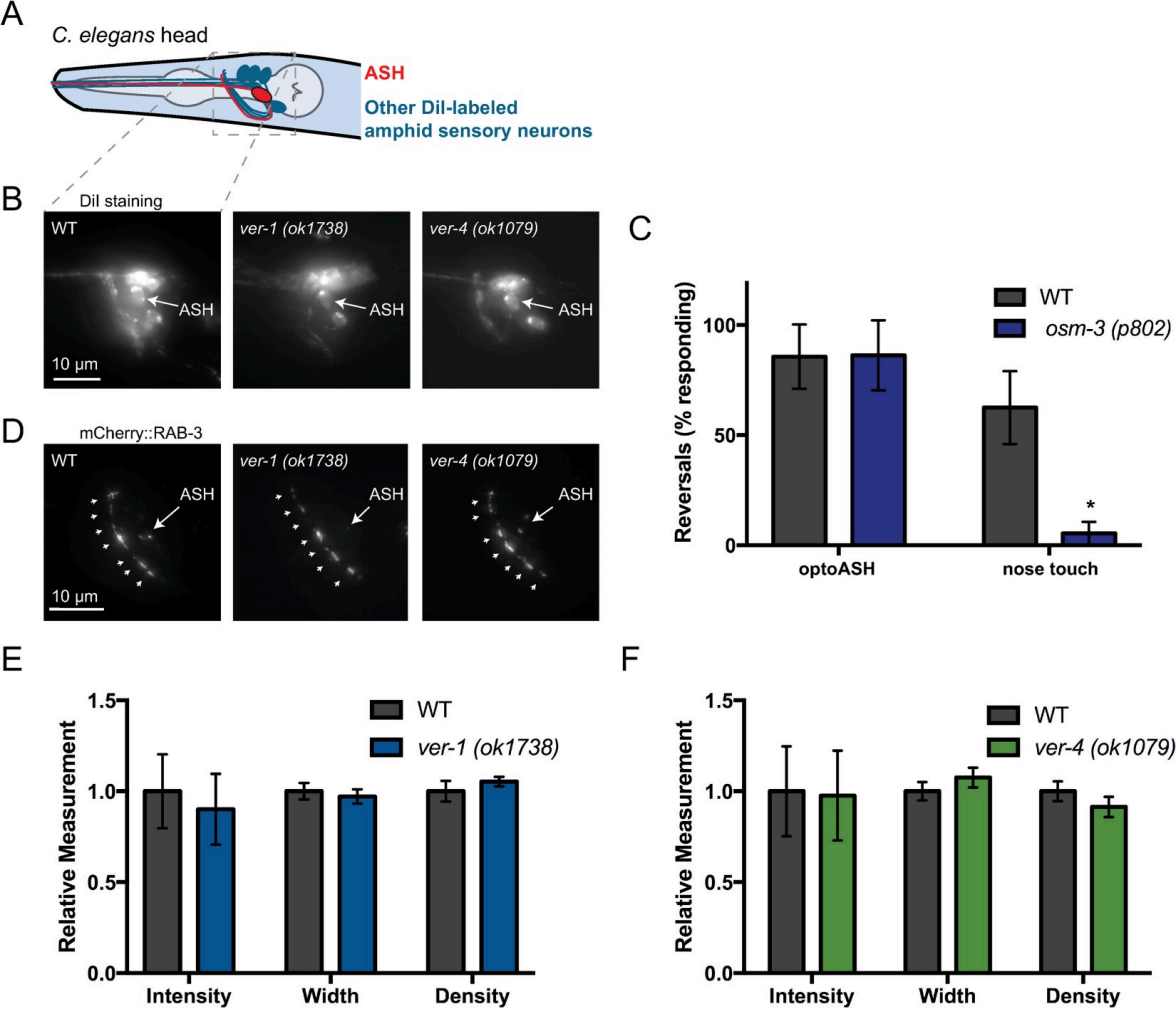
ASH development and synaptic vesicle clustering appear normal in *ver-1* and *ver-4* mutants. (A) Schematic drawing of the *C*. *elegans* head showing ASH (red) and other amphid sensory neurons (blue) that are labeled upon incubation with the lipophilic fluorescent dye DiI. Dashed box represents imaged nerve ring (NR) region for (B,D). (B) Representative images of DiI-labeled sensory neurons in WT and *ver* mutants. Note labeled ASH neurons. (C) Response of *osm-3* mutant worms with known defects in ASH dendrite development to OptoASH and nose touch stimulation. Mean ± SD are shown (n ≥ 13 worms). Values that differ significantly from wild type are indicated by asterisks above each bar. *p ≤ 0.05, Student’s *t* test. (D) Representative images of mCherry::RAB-3 puncta in the NR of WT and *ver* mutants. Arrowheads indicate nerve ring synaptic puncta. The ASH soma is indicated by an arrow. (E,F) Quantification of the intensity, width, and density of mCherry::RAB-3 puncta in the nerve ring of WT and *ver* mutants. Mean ± SEM are shown. (n ≥ 14 worms from 3 experiments).

In agreement with a likely postsynaptic site of action in GLR-1-expressing interneurons, we observed no obvious defects in ASH sensory neurons in *ver* mutants. ASH dendrite elongation and sensory cilia development, as measured by backfilling the ASH soma and dendrite with the lipophilic dye DiI, appeared grossly normal in *ver* mutants (Fig 2A and 2B). Mutants with defects in ASH cilia development, such as *osm-3* mutants, are nose touch defective [49,50] but have normal optoASH responses (Figs 2C and S4)[39]. In contrast, loss of *ver-1* or *ver-4* results in defects in the optoASH response (Fig 1A), suggesting that the *ver* defect lies downstream of ASH sensory transduction and activation. Consistent with a postsynaptic site of action, we found no alteration in the levels or distribution of the synaptic vesicle-associated protein RAB-3 in ASH axons in the nerve ring of *ver-1* or *ver-4* mutants compared to controls (Fig 2D–2F and S2 and S3 Tables). Although other neurons in addition to ASH contribute to the nose-touch response [27,30], these results, together with our rescue data, suggest that the VERs act in GLR-1-expressing neurons downstream of ASH activation.

To test whether *ver* mutants have a more global defect in glutamate signaling rather than a specific defect in ASH-mediated signaling, we analyzed another GLR-1-mediated behavior: spontaneous locomotion reversals. The frequency of spontaneous reversals correlates with the strength of glutamate signaling. Mutants with systemic defects in glutamatergic signaling such as *eat-4* [51,52], *glr-1* [35,53], or mutants with reduced synaptic GLR-1 levels or function [10,41,54–56] reverse less frequently. Conversely, mutants with increased GLR-1 signaling, such as animals expressing a constitutively active version of GLR-1 (GLR-1(A/T))[52] or mutants with greater levels of synaptic GLR-1 [57,58] reverse more frequently. Although worms lacking ASH are nose touch defective [27], they exhibit normal spontaneous reversals [59], indicating that spontaneous reversal defects cannot be attributed to a selective impairment in ASH-dependent glutamate signaling. We found that *ver-1* and *ver-4* mutants have reduced reversal frequencies compared to controls and that the defect was comparable to that observed in *glr-1* mutants (Figs 1E and S3). We also found that mutations in *ver-1* and *ver-4* suppress the increased reversal frequency observed in animals expressing GLR-1(A/T) (Figs 1F and 1G and S3). Because effects of GLR-1(A/T) are independent of presynaptic glutamate release [52], these data reinforce the idea that *ver* mutant defects are unlikely due to impaired presynaptic glutamate release. Collectively, our data suggest that *ver* mutants do not have a specific defect in ASH-dependent glutamatergic function, but rather a global postsynaptic defect in GLR-1-mediated glutamatergic signaling.

### VER-1 and VER-4 act in GLR-1-expressing neurons to promote cell surface levels of GLR-1

We next tested whether *ver-1* or *ver-4* affect the expression or trafficking of GLR-1 receptors themselves. Analysis of a functional GLR-1::GFP (expressed with *glr-1p*) at postsynaptic sites in the ventral nerve cord (VNC) [51,60] revealed a small decrease in GLR-1::GFP puncta fluorescence intensity accompanied by a slight increase in size (measured by puncta width) in *ver-1* and *ver-4* mutant worms (S5A–S5C and S6 Figs and S4 and S5 Tables). In contrast, we observed no change in the intensity, size, or density of the presynaptic marker synaptobrevin (SNB-1::GFP) in the VNC (S5D–S5E Fig and S6 Table), suggesting that changes in GLR-1 puncta observed in *ver* mutants are specific and unlikely due to gross defects in synapse development.

**Fig 3:**
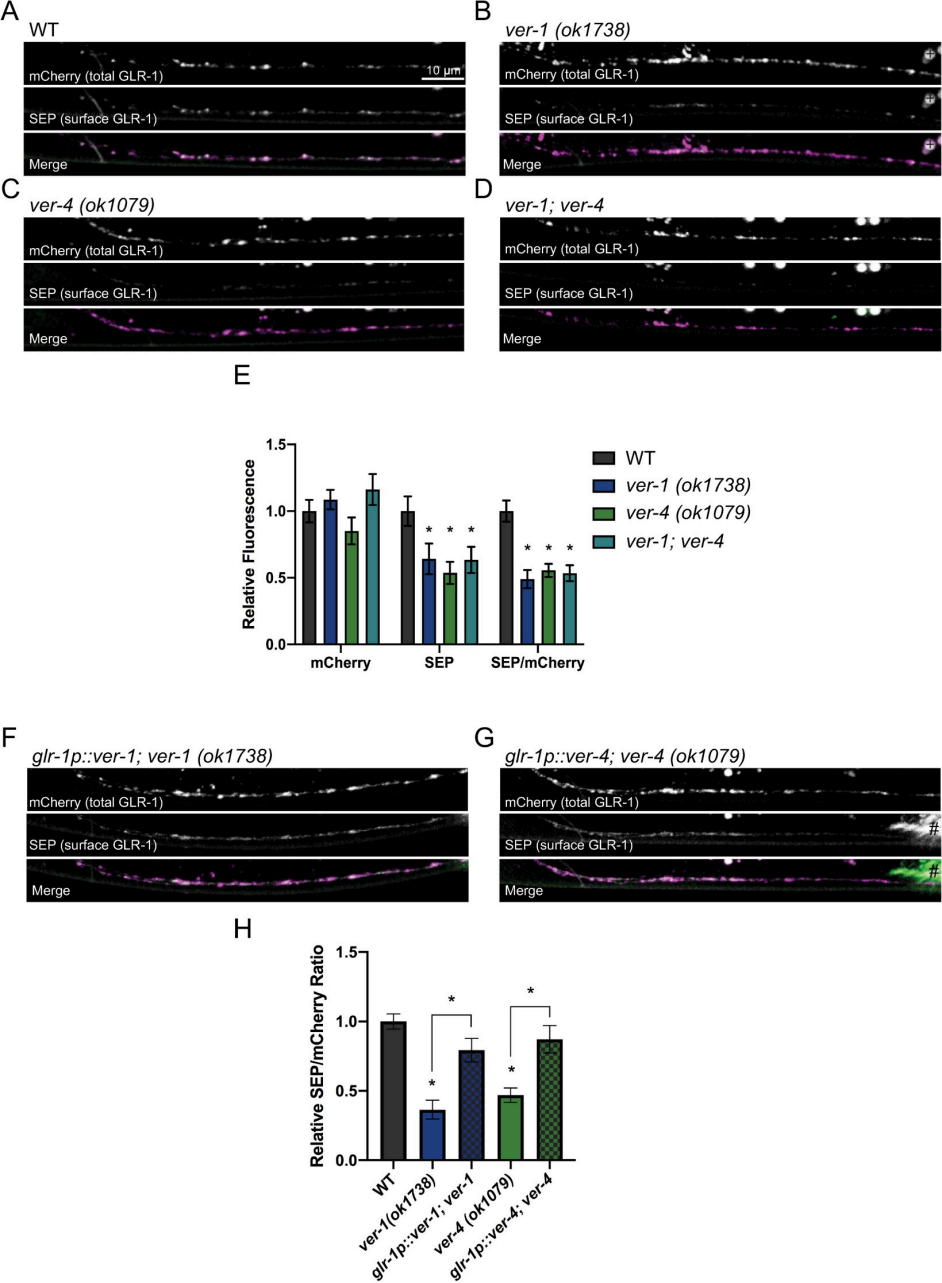
VER-1 and VER-4 act in GLR-1-expressing neurons to promote cell surface levels of GLR-1. (A-D) Representative images of SEP::mCherry::GLR-1 in the anterior VNC of AVA in the indicated genotypes are shown. (E) Quantification of SEP (surface GLR-1) and mCherry (total GLR-1) fluorescence and the SEP/mCherry ratio of genotypes shown in A-D. (F-H) Representative images and quantification of the SEP/mCherry GLR-1 ratio for the indicated genotypes are shown. # marks fluorescence from a co-injection marker. For this and other images, small round circles (marked in B by +) are auto-fluorescent gut granules. Mean ± SEM are shown (n ≥ 28 worms from ≥ 3 experiments). Values that differ significantly from wild type are indicated by asterisks above each bar, whereas other comparisons are marked by brackets. *p ≤ 0.05, ANOVA, Tukey’s multiple comparison test. Merge images have been false colored magenta (mCherry) and green (SEP).

The modest changes in GLR-1::GFP relative to the strong glutamatergic behavioral defects prompted us to test if the subcellular distribution of GLR-1 between surface and internal compartments was altered in *ver* mutants. We analyzed GLR-1 distribution using a previously described transgenic strain that expresses a functional GLR-1 tagged at the N-terminus with mCherry and pH-sensitive superecliptic pHluorin (SEP) (SEP::mCherry::GLR-1) in AVA interneurons [61,62]. AVA is a GLR-1-expressing command interneuron that plays a major role in reversal behavior [36,37,52,59]. SEP fluorescence is quenched in the acidic endosome environment and thus represents an estimate of cell surface GLR-1 [63–65], while mCherry fluoresces in both compartments and thus represents an estimate of total GLR-1 (i.e., surface and internal pools) (S7A and S8 Figs). We found reduced SEP fluorescence (surface GLR-1) levels in the VNC of *ver-1* and *ver-4* mutants (Figs 3A–3C and 3E and S9). This reduction in cell surface GLR-1 is not due to altered GLR-1 expression as we observed similar levels of mCherry fluorescence (total GLR-1) in the VNC (Fig 3A–3C and 3E) and soma (S7B–S7C and S8 Figs) of wild type, *ver-1*, and *ver-4* mutants. Furthermore, expression of wild-type *ver-1* or *ver-4* cDNA in GLR-1-expressing neurons rescued the decreased SEP fluorescence observed in their respective mutant backgrounds (Figs 3F–3H and S9). Finally, we found that *ver-1;ver-4* double mutants exhibit reduced GLR-1 surface levels indistinguishable from either single mutant (Fig 3D and 3E), consistent with the non-additive effects of *ver-1* and *ver-4* mutations on behavior (Fig 1B). Together, these results show that VER-1 and VER-4 act together in GLR-1-expressing neurons to promote cell surface levels of GLR-1.

### The VER ligand PVF-1 promotes cell surface levels of GLR-1 and glutamatergic behavior

**Fig 4:**
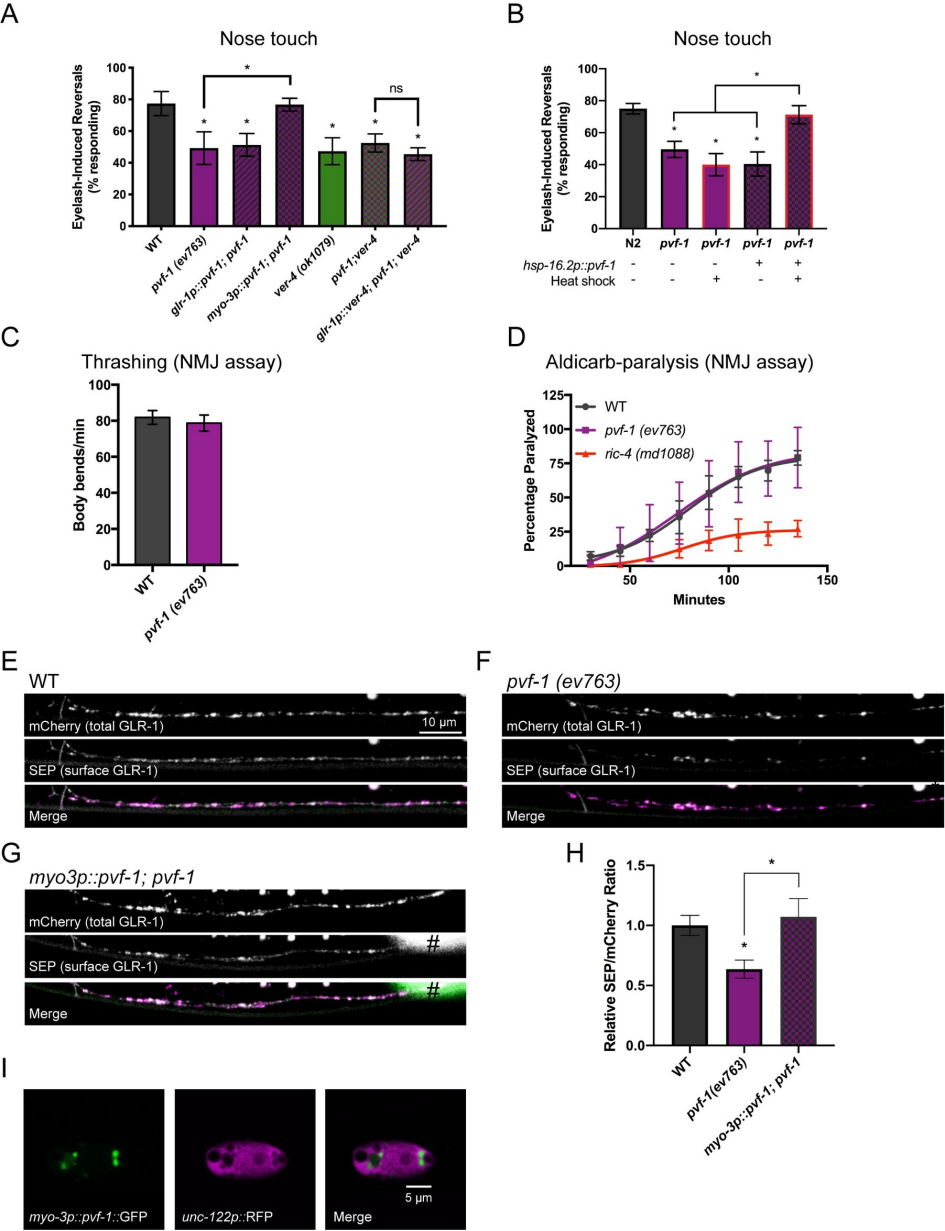
The VER ligand PVF-1 is required for proper GLR-1 trafficking and behavior. (A) Nose touch responses for the indicated mutants and transgenic strains are shown. (B) Nose touch responses for WT worms and *pvf-*1 mutants with and without a 30°C heat shock for 3 hours and/or *hsp-16*.*2*::*PVF-1* rescue transgene as indicated. Rates of thrashing (C) and aldicarb-induced paralysis (D) are shown. Mean ± SEM are shown (n ≥ 20 worms from 3 experiments). Values that differ significantly from wild type are indicated by asterisks above each bar, whereas other comparisons are marked by brackets. *p ≤ 0.05 ANOVA, Dunnett’s multiple comparison test. Representative images (E-G) and quantification (H) of the SEP/mCherry ratio for the indicated genotypes are shown. # marks fluorescence from a co-injection marker. Mean ± SEM are shown (n ≥ 23 worms from 3 experiments). Values that differ significantly from wild type are indicated by asterisks above each bar, whereas other comparisons are marked by brackets. *p ≤ 0.05, ANOVA, Tukey’s multiple comparison test. (I) Confocal image showing the accumulation of PVF-1::GFP in vesicles inside a coelomocyte (marked by *unc-122p*::*RFP*). Merge images have been false colored magenta (mCherry) and green (SEP).

The *C*. *elegans* genome encodes one putative VER ligand, PVF-1, that is homologous to and functionally conserved with mammalian VEGF [23,24]. We tested whether PVF-1 acts with the VERs to regulate GLR-1 trafficking and glutamate-dependent behavior. Similar to *ver-1* and *ver-4* mutants, *pvf-1 (ev763)* loss of function mutants had defects in the nose touch response (Figs 4A and S10). The *pvf-1 (ev763)* allele is likely to be null as it contains a 1465 bp deletion that eliminates the start codon, exons 1–2 and part of exon 3 [23]. Because *pvf-1* expression has only been detected in body wall muscle [23], we tested if muscle expression of *pvf-1* was sufficient to rescue the *pvf-1* mutant defect in the nose touch response. We found that this defect could be rescued by expression of wild-type *pvf-1* cDNA in body wall muscle (using the *myo-3p* promoter), but not in GLR-1-expressing neurons (using the *glr-1* promoter). This data is intriguing as it suggests that PVF-1 function may be dependent on the cell type from which it is expressed and secreted.

Genetic analysis showed that *pvf-1;ver-4* double mutants displayed reduced nose touch responses that were not significantly different from *pvf-*1 (p = 0.99) or *ver-*4 (p = 0.97) single mutants (Fig 4A). This data suggests that *pvf-1* and *ver-4* act in the same pathway to regulate glutamate signaling, consistent with a prior study showing that PVF-1 functions with the VERs to control male tail development [23]. Similar to *ver-1* and *ver-4* mutants, *pvf-1* mutants had normal NMJ function as determined by the rates of thrashing and aldicarb-induced paralysis (Figs 4C and 4D and S10 and S7 Table). In addition, although expression of *ver-4* in GLR-1-expressing neurons could rescue the nose touch defect observed in *ver-4* mutants (Fig 1B), the same *ver-4* transgene could not rescue in the absence of *pvf-1* in *pvf-1;ver-4* double mutants (Fig 4A). These data suggest that VER-4 function in GLR-1-expressing neurons requires endogenous PVF-1.

We next tested whether heat-shock inducible expression of wild-type *pvf-*1 cDNA after the vast majority of glutamatergic synapse development is thought to be complete [66] was able to rescue the *pvf-1* mutant defect in glutamatergic nose-touch behavior. Control or *pvf-1* mutant worms were raised at 20°C until the fourth larval (L4) stage, and then heat shocked at 30°C for 3 hours to induce expression of PVF-1. Worms were subsequently shifted back to 20°C to recover and assayed for their nose-touch responses as adults. We found that inducible expression of PVF-1 after the L4 stage was sufficient to rescue the *pvf-1* mutant nose touch defect (Figs 4B and S10). No rescue was observed in *pvf-1* mutants carrying the inducible transgene (*hsp-16*.*2p*::*pvf-1)* in the absence of the heat-shock treatment, nor in *pvf-1* mutants that were heat-shocked in the absence of the inducible transgene (Fig 4B). These data indicate that PVF-1 may act in the mature nervous system to regulate glutamatergic signaling.

Analysis of the subcellular distribution of GLR-1 revealed that *pvf-1* mutants also exhibited a specific reduction in the surface pool of GLR-1 in AVA command interneurons (Figs 4E,4F and 4H and S10), similar to the decrease observed in *ver-1* and *ver-4* mutants. Expression of PVF-1 in body wall muscle (using *myo-3p*) rescued the reduced levels of surface GLR-1 in *pvf-1* mutants (Fig 4G and 4H). Finally, we confirmed that GFP-tagged PVF-1 (under control of the *myo-3p*) was being secreted from muscle based on the accumulation of PVF-1::GFP in coelomocytes (marked by *unc-122p*::*RFP*)(Fig 4I). Accumulation of fluorescently-tagged proteins in coelomocytes, which are specialized scavenger cells that endocytose and degrade secreted proteins from the extracellular pseudocoelomic space [67], has previously been used to monitor neuropeptide secretion from neurons [68]. Together, these results indicate that loss of the ligand *pvf-1* or receptors *ver-1* or *ver-4* result in similar defects in cell surface GLR-1 and glutamatergic behavior, suggesting that PVF-1/VER signaling acts to control GLR-1 trafficking and glutamate signaling. These data also suggest that PVF-1 is secreted from muscle, is required for VER function in GLR-1-expressing neurons, and can function in the mature nervous system to regulate glutamatergic signaling.

Because a previous study in mammalian neurons showed that co-application of exogenous VEGF and NMDA could increase surface levels of AMPARs in cultured rodent neurons [21], we tested if GLR-1 surface levels were altered in *nmr-1*/NMDAR subunit NR1 loss of function mutants. We found no change in the surface levels of GLR-1 in *nmr-1 (ak4)* mutants (GLR-1 SEP/mCherry ratio (Norm.) Mean±SEM: WT: 1.0±0.11, n = 24 animals; *nmr-1 (ak4)*: 0.87±0.1, n = 28 animals, n.s. vs WT, p = 0.87). This data, together with prior data from the Maricq lab showing that *nmr-1* mutants have wild-type nose touch responses [35], suggest that unlike *pvf-1* and the *vers*, *nmr-1* is not required for normal surface levels of GLR-1 or glutamatergic nose touch behavior. It is therefore unlikely that reduced GLR-1 surface levels we observed in *pvf-1/ver* mutants is mediated by changes in NMR-1 signaling.

### PVF-1/VER signaling functions with the VPS-35/retromer recycling pathway to promote cell surface levels of GLR-1

**Fig 5:**
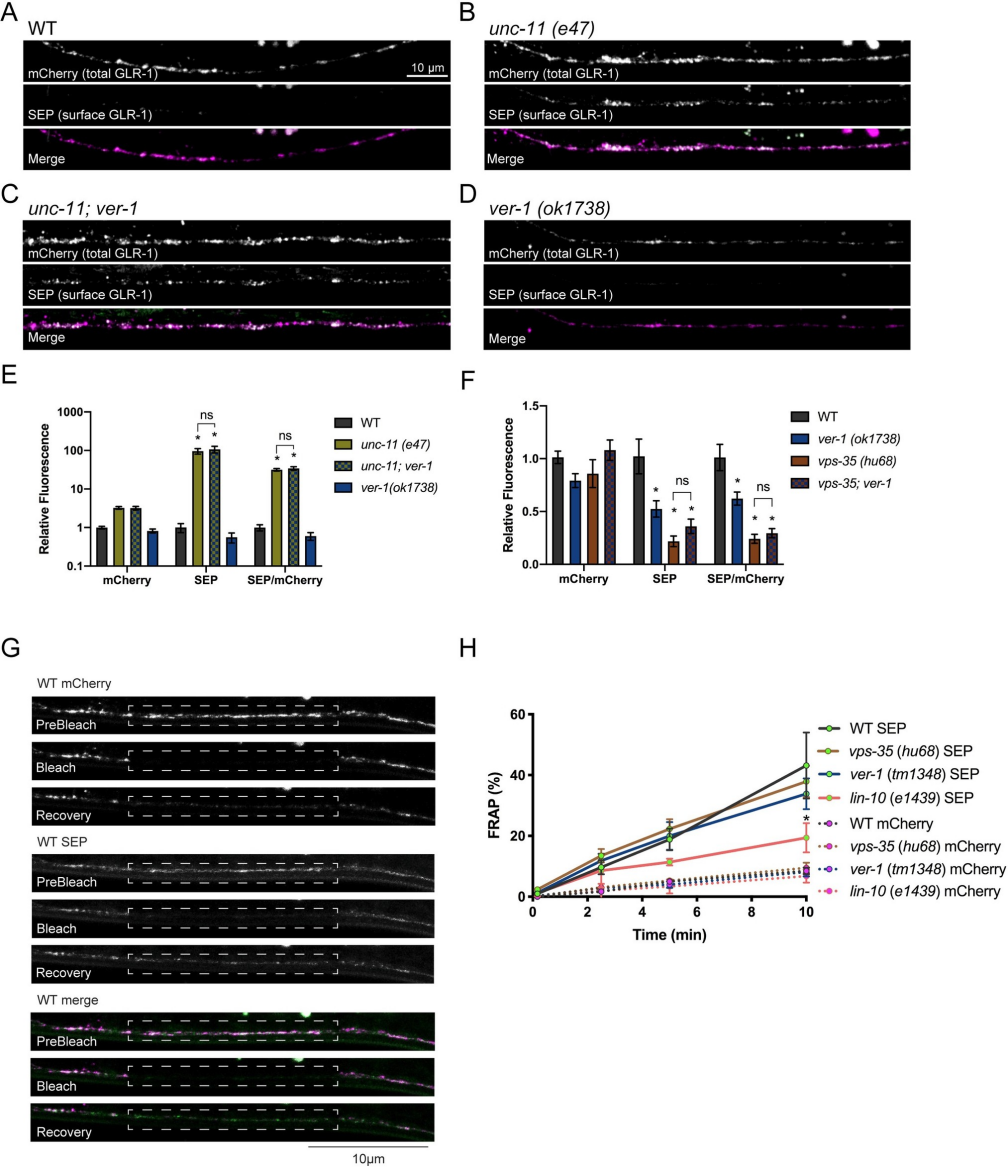
PVF-1/VER signaling acts with the VPS-35/retromer recycling pathway to regulate GLR-1 surface levels. (A-E) Representative images and quantification of the SEP and mCherry fluorescence and the SEP/mCherry ratio of the indicated genotypes are shown. Images were acquired at low laser intensity to avoid saturation. Note Y-axis log scale. Mean ± SEM are shown (n ≥ 20 worms from 3 experiments). *p ≤ 0.05 ANOVA followed by Tukey’s multiple comparison test. (F) Quantification of the SEP and mCherry fluorescence or the SEP/mCherry ratio of the indicated genotypes are shown. Mean ± SEM are shown (n ≥ 30 worms from ≥ 3 experiments). *p ≤ 0.05, ANOVA, Tukey’s multiple comparison test. Values that differ significantly from wild type are indicated by asterisks above each bar, whereas other comparisons are marked by brackets. (G) Representative Dual FRAP images showing pre-bleach, bleach, and 10 minute recovery images of SEP::mCherry::GLR-1 (*akIs201*) in a region (outlined by a dotted line) in the VNC of L4 stage larval wild type worms. (H) Quantification of Dual FRAP (bleach at 0 min) of SEP∷mCherry∷GLR-1 in the VNC of WT, *ver-1*, *vps-35* or *lin-10* worms. A 50% smaller ROI that was centered within the larger bleach ROI shown was used for quantification to minimize contributions from laterally diffusing receptors. At 10 min, *lin-10* SEP vs WT SEP, *p < 0.001. Two-way repeated measures ANOVA, followed by Tukey’s multiple comparison test. Merge images have been false colored magenta (mCherry) and green (SEP).

We next investigated the mechanism by which the VERs regulate surface levels of GLR-1. Several trafficking pathways contribute to AMPAR levels in the postsynaptic membrane including delivery from the soma, lateral diffusion, and endocytosis followed by degradation or recycling to the cell surface [3]. To determine whether the VERS act upstream or downstream of GLR-1 endocytosis in the VNC, we analyzed *ver-1;unc-11* double mutants. The clathrin adaptin UNC-11/AP180 is required for endocytosis of GLR-1 in the VNC and GLR-1 accumulates at the cell surface in *unc-11* mutants [51]. If the reduced cell surface pool of GLR-1 seen in *ver-1* mutants was the result of an inability to deliver newly synthesized GLR-1 from the soma to the plasma membrane, we would expect *ver-1;unc-11* double mutants to appear similar to *ver-1* single mutants. In contrast, we found that *ver-1;unc-11* double mutants had an increase in SEP fluorescence levels (surface GLR-1) in the VNC indistinguishable from *unc-11* single mutants (Figs 5A–5E and S11). These data suggest that, in the wild-type context, VER-1 does not regulate anterograde trafficking or initial plasma membrane insertion of GLR-1 upstream of clathrin-mediated endocytosis. Instead, our data support a role for the VERs in promoting cell surface levels of GLR-1 by either inhibiting GLR-1 endocytosis or increasing GLR-1 recycling from internal compartments.

Previous studies in *C*. *elegans* [10] and in mammalian neurons [8,9,11], showed that the retromer endosomal sorting complex mediates AMPAR recycling to the plasma membrane. Retromer is a multi-subunit complex consisting of VPS-26, VPS-29, VPS-35 and sorting nexins that assembles on endosomes and mediates retrograde recycling via the Golgi [69]. Interestingly, the *ver* mutants exhibited defects in GLR-1 trafficking and behavior reminiscent of *vps-35* retromer mutants. For example, *vps-35* mutants have reduced levels of GLR-1 in the VNC, and decreased glutamatergic nose touch responses [10]. Furthermore, *vps-35* and other retromer components act downstream of endocytosis in the VNC to control GLR-1 recycling. Thus, we examined whether the VERs act together with retromer to promote GLR-1 trafficking. Consistent with the model proposed by Zhang et al. [10], we found that *vps-35 (hu68)* mutants have reduced SEP fluorescence levels (surface GLR-1) in the VNC (Figs 5F and S11). The *vps-35 (hu68)* allele is likely a null allele as it contains a deletion that starts in the middle of exon 2 and ends in the 7^th^ and last intron, resulting in the deletion of almost the entire coding region of the protein [70]. Interestingly, this reduction in surface GLR-1 was not enhanced in *vps-35;ver-1* double mutants, suggesting that VER-1 and VPS-35 act in the same pathway to promote cell surface levels of GLR-1.

Retromer was originally shown to mediate retrograde transport of cargo from endosomes back to the Golgi in yeast [71] and soma in mammalian neurons [72,73]; however, a recent study showed that retromer can also act in mammalian dendrites to regulate local recycling and insertion into the plasma membrane [8]. Dual fluorescence recovery after photobleaching (FRAP) of SEP::mCherry::GLR-1 has previously been used to monitor synaptic delivery and local insertion of GLR-1 in the VNC [62]. Thus, we used FRAP to test if *vps-35* regulates local insertion of GLR-1 in the VNC. As previously described, the mCherry signal is photobleached on intracellular and surface GLR-1 receptors, whereas the SEP signal is only photobleached on surface GLR-1 because the quenched SEP in low pH endosomes is resistant to photobleaching [61,74]. We found that recovery of SEP fluorescence (surface GLR-1) after photobleaching was unaltered in *vps-35* mutants compared to wild type (Fig 5G–5H and S8 Table), suggesting that local recycling and membrane delivery of endosomal GLR-1 in the VNC does not require *vps-35*/retromer. These data are consistent with a previous study in *C*. *elegans*, which showed that retromer promotes long-range retrograde recycling of GLR-1 via the soma [10]. In contrast, we found that loss of function mutations in *lin-10*, a PDZ protein that regulates GLR-1 recycling and surface levels [55], exhibited reduced recovery of SEP fluorescence after photobleaching (Fig 5H), suggesting that the FRAP assay is sensitive enough to detect changes in recycling. Interestingly, we found no change in the rate of SEP fluorescence recovery after photobleaching in *ver-1* mutants compared to WT (Fig 5H), suggesting that like retromer mutants, *ver-1* does not appear to regulate local recycling of GLR-1 in the VNC. Together, these data are consistent with a genetic model in which the VERs act together with retromer and long-range recycling to promote surface levels of GLR-1 at synapses.

## Discussion

Extrasynaptic secreted factors, such as growth factors, hormones and neuropeptides, modulate synaptic signaling, circuit function and behavior. However, identification of the relevant factors and understanding how they regulate synapses and behavior is far from complete. In this study, we identified the secreted ligand PVF-1 and the VEGFR homologs *ver-1* and *ver-4* as critical regulators of GLR-1 surface levels and glutamate-dependent behaviors in *C*. *elegans*.

To screen for genes involved in mediating glutamate-dependent locomotor reversal responses we used optogenetic activation of ASH sensory neurons. The ASH neuron pair are polymodal sensory neurons that can trigger reversals when activated by multiple stimuli including gentle nose touch and high osmolarity [27,33,34]. We identified *ver-1* and *ver-4* in this optogenetic screen using a one-second photostimulation paradigm modeled after Schmitt et al. (2012) [75] and subsequently showed that *ver-1* and *ver-4* loss of function mutants have strong defects in the nose touch response (Fig 1). Previous studies using a two-second photostimulation paradigm found that optogenetic activation of ASH-mediated reversal behavior more closely mimics ASH activation by high osmolarity [76–78]. Since ASH likely uses graded neurotransmission [40] to encode differences in the modality of sensory stimulation, with nose touch leading to less glutamate release than exposure to high osmolarity [35], it is possible that our photostimulation conditions may more closely simulate nose touch. Nevertheless, it will be interesting to test if the *ver* mutants show altered responses to other aversive stimuli detected by ASH.

Interestingly, *ver-1* and *ver-4* mutants appear to have stronger effects on glutamatergic behavior (Fig 1) than on GLR-1 surface levels (Fig 3). While we do not know the reason for this difference, one possibility is that the *ver* mutants may affect the trafficking or activity of other critical proteins required for GLR-1 function such as the auxiliary subunits SOL-1 or STG-2 [54,79]. Alternatively, the residual surface GLR-1 detected in *ver-1* and *ver-4* mutants may not be precisely localized at the synapse.

There are four genes in the VER family in *C*. *elegans*. *ver-1*, *ver-2* and *ver-3* have been reported to be expressed in non-overlapping cells of neural origin based on promoter::GFP reporters [22]. *ver-1* is expressed in glia-like sheath cells of amphid and phasmid neurons, *ver-2* is expressed in the chemosensory neuron ADL, and *ver-3* is expressed in ALA neurons [22]. Although this implies that the *vers* may have relatively restricted expression patterns, subsequent analysis of *ver* mutant phenotypes and expression based on large-scale transcriptome studies hint that the *ver* genes may have broader functions than previously recognized. Two studies identified functions for the *vers* in processes that were consistent with roles in other cell types. Lemieux et al. (2013), showed that *ver-2* and *ver-3* mutants have defects in pharyngeal pumping rates [80], and Dalpe et al. (2013) found that all four *ver* genes regulate male tail development in combination with semaphorin/plexin [23]. In the latter study, expression of *ver-1* or mammalian VEGFR1 or VEGFR2 in ray neurons and the ray structural cell (using the *lin-32* promoter) was sufficient to rescue the male tail defect. Given the role of all four *ver* genes in male tail development, it will be interesting in the future to test if *ver-2* and *ver-3*, and other genes known to interact with VEGFRs like semaphorins and plexins, affect GLR-1 trafficking and glutamatergic behaviors.

Several large-scale transcriptome studies also suggest that the *ver* genes may act in more cell types than those originally identified by Popovici et al. (2002) [22]. In particular, *ver-1*, *ver-2* and *ver-4* [81] and *ver-3* [82] transcripts were detected in *glr-1*-expressing neurons including the command interneurons, and another study found that *ver-4* transcripts were present at low levels in a subset of interneurons that also expressed *glr-1* [83]. Since these expression patterns have not yet been confirmed, we cannot formally rule out an indirect role for VER-1 and VER-4 in non-GLR-1-expressing cells. However, we favor a model where VER-1 and VER-4 act in GLR-1-expressing neurons to regulate surface levels of GLR-1 and glutamatergic behavior for several reasons. First, mammalian VEGFRs are expressed broadly in the nervous system including in AMPA receptor-expressing hippocampal neurons [18–21] and their expression can be upregulated by external stimuli [16]. Similarly in *C*. *elegans ver-1* expression is weak in non-dauers and dramatically upregulated in amphid glia during dauer development [22,25]. Thus, it would not be surprising if the *ver* genes are normally expressed at low levels in the nervous system and upregulated under certain conditions or in response to specific stimuli. Second, we found that *ver-1* and *ver-4* mutants had reduced cell surface levels of GLR-1 in AVA command interneurons and defects in two glutamatergic behaviors, the nose-touch response and spontaneous reversal behavior (Figs 1 and 3). Third, *glr-1 ver-1;ver-4* triple mutants had defects in nose touch behavior that was non-additive, suggesting that the *vers* act in the same pathway as *glr-1* (Fig 1B). Fourth, expression of *ver-1* or *ver-4* in GLR-1-expressing neurons rescued the nose-touch defects and reduced surface levels of GLR-1 observed in *ver-1* and *ver-4* mutants, respectively (Figs 1 and 3). Finally, while expression of *ver-4* in GLR-1-expressing neurons rescued the nose-touch defect in *ver-4* single mutants, it did not rescue in *ver-4;pvf-1* double mutants (Fig 4A), suggesting that VER-4 function in GLR-1-expressing neurons is dependent on endogenous PVF-1.

PVF-1, the only known VEGF homolog in *C*. *elegans*, is structurally and functionally conserved with mammalian VEGF. *C*. *elegans* PVF-1 can bind and activate mammalian VEGFRs to induce tube formation in human endothelial cells and promote angiogenesis in chick embryos [24]. PVF-1 has been shown to be expressed in body wall muscle based on a *pvf-1* transcriptional reporter and immunohistochemical staining using an anti-PVF-1 antibody [23]. We found that *pvf-1* mutants had defects in the nose touch response that could be rescued with expression of PVF-1 in muscle, but not in GLR-1-expressing neurons (Fig 4A). This data is intriguing because it suggests that even though PVF-1 is a secreted protein, it only appears to be functional when expressed from certain cells. We also found that *pvf-1* mutants had decreased surface levels of GLR-1, and expression of PVF-1 in muscle rescued the defect (Fig 4H). We propose that PVF-1 may only be active after being modified or processed in muscle, or that PVF-1 activity may require another factor that is co-released from muscle. Together, our data support a model where PVF-1 is expressed and secreted from body wall muscle which then activates VER-1 and VER-4 in GLR-1-expressing neurons to promote surface levels of GLR-1 and glutamatergic behavior. In addition, our genetic data are consistent with VER-1 and VER-4 acting together with the retromer recycling complex to promote long-range recycling of GLR-1.

Although the stimuli or conditions that regulate PVF-1/VER signaling are not known in *C*. *elegans*, there are many stimuli known to increase VEGF expression in mammals. For example, hypoxia is a well-known stimulus that increases expression of VEGF to promote angiogenesis [84]. We considered the possibility that hypoxia may increase PVF-1/VER signaling to promote surface levels of GLR-1 and glutamatergic signaling in the worm. However, in *C*. *elegans*, hypoxia is known to have the opposite effect on GLR-1, resulting in decreased surface levels and reduced glutamatergic signaling [85]. Thus, hypoxia is unlikely to induce expression of PVF-1 as a mechanism to promote GLR-1 surface levels and glutamatergic signaling in *C*. *elegans*.

We speculate that muscle cells could alter PVF-1 expression, secretion or activity in response to repeated activation of motor programs that result in backward movement. Because motor neurons release neuropeptides in addition to neurotransmitters [86], it is possible that the frequent signaling by DA and VA motor neurons responsible for backward movement [37,87] could ultimately decrease PVF-1 activity. Based on our findings that *pvf-1* and *ver* mutants have reduced surface GLR-1 and reversal responses, a reduction in PVF-1 secretion, for example, would be predicted to decrease the responsiveness of GLR-1-expressing interneurons to stimuli that would normally trigger reversal behavior. Alternatively, such repeated activation of backward motor neurons could increase the secretion of a molecule that antagonizes PVF-1/VER signaling. Either way, this feedback mechanism could ultimately lead to habituation of *C*. *elegans* to surrounding aversive stimuli and promote forward dispersal to a more favorable environment.

In conclusion, our results suggest a model whereby PVF-1 is expressed and released from muscle resulting in activation of VER-1 and VER-4, which in turn promote increased surface levels of GLR-1 in interneurons and glutamatergic behavior. VEGF can enhance hippocampal LTP and memory in rodents [17,20], consistent with a conserved function for VEGF in regulating the nervous system and glutamatergic behavior from worms to mammals. Our study provides in vivo genetic evidence that PVF-1/VER signaling promotes surface levels of GLR-1 to control behavior. Intriguingly, chemical LTP can stimulate VEGF release from hippocampal slices [21]. Future experiments will be focused on identifying stimuli, such as changes in neuronal or muscle activity, that alter PVF-1/VER expression or signaling.

## Materials and methods

### Worm strains

All strains were maintained at 20°C as described previously [88]. N2 Bristol was used as the wild-type strain. The *pvf-1 (ev763)* allele contains a 1465 bp deletion that eliminates the start codon, exons 1–2 and part of exon 3. It is likely to be a null [23]. The *ver-1 (ok1738)* allele contains an in-frame deletion that removes part of the extracellular region (amino acids 168–417) including Ig domains 2 and 3 [25], which based on homology to mammalian VEGFRs are predicted to bind ligand [43,44]. Thus, *ver-1 (ok1738)* is likely unable to bind ligand and therefore represents a functional null for receptor signaling. The *ver-1 (tm1348)* allele contains a frame-shift deletion predicted to result in a truncated protein of 267 amino acids that lacks the transmembrane and intracellular kinase domains [25]. The *ver-4 (ok1079)* allele contains a ~1kb deletion that starts at nucleotide 1095/1096 after Lys365 and ends at nucleotide 1940/1941. This deletion is predicted to disrupt extracellular Ig domains 4–6, which are required for VEGFR dimerization and activation [45], resulting in a frameshift and premature stop codon 12 nucleotides downstream of the deletion. Thus, the *ver-4 (ok1079)* allele is predicted to encode a truncated 365 amino acid protein comprised of the first three N-terminal Ig domains of the extracellular domain but lacking the other 4 Ig, transmembrane and intracellular domains. The *unc-11 (e47)* allele is a null allele and contains a small deletion resulting in a frame-shift and premature stop codon that if translated would result in a 10 amino acid protein [89]. The *vps-35 (hu68)* allele contains a deletion that starts in the middle of exon 2 and ends in the 7^th^ and last intron. It deletes almost the entire coding region of the protein and is thus a likely null allele [70].

Strains used in this study are listed below:

FJ1300: *lin-35 (n745) nuIs25 (glr-1p*::GLR-1∷GFP, *lin-15)* I, *sid-1(pk3321)*, *uIs69 (myo-2p*::mCherry, *unc-119p*::*sid-1)* V;*lite-1 (ce314)*, *ljIs114 (gpa-13p*::FLPase, *sra-6p*::*FTP*::ChR2::YFP) X

AQ2235: *lite-1 (ce314)*, *ljIs114 (gpa-13p*::FLPase, *sra-6p*::*FTP*::ChR2::YFP) X [38]

FJ1282: *eat-4 (n2474)* III;*lite-1 (ce314)*, *ljIs114 (gpa-13p*::FLPase, *sra-6p*::*FTP*::ChR2::YFP) X

FJ1281: *glr-1 (n2461)* III;*lite-1 (ce314)*, *ljIs114 (gpa-13p*::FLPase, *sra-6p*::*FTP*::ChR2::YFP) X

FJ1306: *usp-46 (ok2232)* III*;lite-1 (ce314)*, *l ljIs114 (gpa-13p*::FLPase, *sra-6p*::*FTP*::ChR2::YFP) X

FJ1426: *osm-3 (p802)* IV*;lite-1 (ce314)*, *ljIs114 (gpa-13p*::FLPase, *sra-6p*::*FTP*::ChR2::YFP) X

KP4: *glr-1 (n2461)* III

PR802: *osm-3 (p802)* IV

VC1263: *ver-1 (ok1738)* III

VC40144: *ver-1 (gk483430)* III

FX01348: *ver-1 (tm1348)* III

RB1100: *ver-4 (ok1079)* X

FJ1467: ver-1 (ok1738) III;ver-4 (ok1079) X

ESL17: glr-1 (n2461) ver-1 (ok1738) III;ver-4 (ok1079) X

*VC30127*: *ver-4 (gk963311)* X

EV763 *pvf-1 (ev763)* III

FJ1606: *pxEx425 (glr-1p*::PVF-1*; unc-122p*::GFP*);pvf-1 (ev763)* III

ESL15: *pzEx423 (myo-3p*∷PVF-1, co-injection marker *unc-122p* ∷GFP*);pvf-1 (ev763)* III

FJ1473: *pvf-1 (ev763)* III*;ver-4 (ok1079)* X

ESL14: *pzEx39*9 *(glr-1p*∷VER-4, co-injection marker *unc-122p* ∷GFP*);pvf-1 (ev763)* III*;ver-4 (ok1079)* X

ESL16: *simEx7 (hsp-16*.*2p*::PVF-1, co-injection marker *unc-122p*::PVF-1*);pvf-1 (ev763) III*

FJ1900: *pzIs44* (*myo-3p*::PVF-1::GFP, co-injection marker *myo-2*::NLS::mCherry*);pzEx500 (unc-122p* ∷RFP)

RM956: *ric-4 (md1088)* V

CB47: *unc-11 (e47)* I

KN555: *vps-35 (hu68)* II

FJ1669: *vps-35 (hu68)* II;*ver-1 (ok1738)* III

KP2006: *nuIs80 (glr-1p*::GLR-1(A/T)∷YFP*)* [41]

FJ1466: *nuIs80 (glr-1p*::GLR-1(A/T)∷YFP*);ver-1 (ok1738)* III

FJ1429: *nuIs80 (glr-1p*::GLR-1(A/T)∷YFP)*;ver-4 (ok1079)* X

FJ1531: *pzEx398 (sra-6p*∷VER-1, co-injection marker *unc-122p* ∷GFP*);ver-1 (ok1738)* III

FJ1492: *pzEx39*0 *(glr-1p*∷VER-1, co-injection marker *unc-122p* ∷GFP*);ver-1 (ok1738)* III

FJ1532: *pzEx39*9 *(glr-1p*∷VER-4, co-injection marker *unc-122p* ∷GFP*);ver-4 (ok1079)* X

FJ601: *pzEx423 (myo-3p*∷PVF-1, co-injection marker *unc-122p* ∷GFP*);pvf-1 (ev763)* III

FJ1499: *akIs201 (rig-3p*::SEP::mCherry::GLR-1*)* V

FJ1547: *akIs201 (rig-3p*::SEP::mCherry::GLR-1*);unc-11 (e47)* I

FJ1512: *akIs201 (rig-3p*::SEP::mCherry::GLR-1*);ver-1 (ok1738)* III

FJ1549: *akIs201 (rig-3p*::SEP::mCherry::GLR-1*);unc-11 (e47)* I*; ver-1(ok1738)* III

FJ1485: *akIs201 (rig-3p*::SEP::mCherry::GLR-1*);ver-4 (ok1079)* X

FJ1534: *akIs201 (rig-3p*::SEP::mCherry::GLR-1*);ver-1 (ok1738)* III*; ver-4 (ok1079)* X

FJ1504: *akIs201 (rig-3p*::SEP::mCherry::GLR-1*);pvf-1 (ev763)* III

FJ1627: *akIs201 (rig-3p*::SEP::mCherry::GLR-1*)*;*lin-10*(e1439) I

FJ1505: *pzEx390 (glr-1p*∷VER-1, co-injection marker *unc-122p* ∷GFP*);akIs201 (rig-3p*::SEP::mCherry::GLR-1*)* V*;ver-1 (ok1738)* III

FJ1538: *pzEx39*9 *(glr-1p*∷VER-4, co-injection marker *unc-122p* ∷GFP*);akIs201 (rig-3p*::SEP::mCherry::GLR-1*)* V*;ver-4 (ok1079)* X

FJ1602: *pzEx423 (myo-3p*∷PVF-1, co-injection marker *unc-122p* ∷GFP*);akIs201(rig-3p*::SEP::mCherry::GLR-1*)* V*;pvf-1 (ev763)* III

FJ464: *pzIs12 (glr-1p*∷HA∷GLR-1∷GFP*)* II [41]

FJ1450: *pzIs12 (glr-1p∷*HA*∷*GLR-1*∷*GFP*)* II*;ver-1 (ok1738)* III

FJ1451: *pzIs12 (glr-1p∷*HA*∷*GLR-1*∷*GFP*)* II*;ver-4 (ok1079)* X

KP2775: *nuIs125 (glr-1p∷*SNB-1∷GFP) [57]

FJ1506: *nuIs125 (glr-1p∷*SNB-1∷GFP*);ver-1 (ok1738)* III

FJ1514: *nuIs125 (glr-1p∷*SNB-1∷GFP*);ver-4 (ok1079)* X

FJ1229: *pzEx337 (sra-6p∷loxSTOPlox∷*mCherry∷ RAB-3, *gpa-13p∷*nCRE, *ttx-3p∷*GFP)

FJ1706: *pzEx337 (sra-6p∷loxSTOPlox∷*mCherry∷ RAB-3, *gpa-13p∷*nCRE, *ttx-3p∷*GFP*);ver-1 (ok1738)* III

FJ1441: *pzEx337 (sra-6p∷loxSTOPlox∷*mCherry∷ RAB-3, *gpa-13p∷*nCRE, *ttx-3p∷*GFP*);ver-4 (ok1079)* X

All tm alleles were kindly provided by the Mitani lab through the National Bio-Resource Project of the MEXT, Japan.

### Molecular biology

Plasmids were generated using standard recombinant DNA techniques, and transgenic strains were created by plasmid microinjection using standard methods. The following plasmids were generated in this study:

***sra-6p*::*ver-1*** (FJ#136) cDNA rescue construct was made by digesting *lin-32p* from pZH260 [23] using NotI and cloning in *sra-6p* amplified from FJ#134 using PCR. FJ#136 was injected at 1 ng/μl with *unc-122p*::*GFP* at 25 ng/μl and pBluescript SK at 74 ng/μl to make *pzEx398*.

***glr-1p*::*ver-1*** (FJ#135) cDNA rescue construct was made by digesting *lin-32p* from pZH260 [23] using NheI and PstI and cloning in *glr-1p* amplified from FJ#127 using PCR. FJ#135 was injected at 1 ng/μl with *unc-122p*::*GFP* at 25 ng/μl and pBluescript SK at 74 ng/ul to make *pzEx390*.

***glr-1p*::*ver-4*** (FJ#137) cDNA rescue construct was made by Bio Basic (Ontario, Canada) by synthesizing *ver-4* and inserting it into pV6 digested with NheI and KpnI. FJ#137 was injected at 10 ng/μl with *unc-122p*::*GFP* at 25 ng/μl and pBluescript SK at 65 ng/μl to make *pzEx399*.

***glr-1p*::*pvf-1*** (FJ#128) was created by amplifying *pvf-1* from N2 cDNA and subcloning it into pV6 using NheI and KpnI. FJ#128 was injected into *pvf-1 (ev763)* mutant worms at 50 ng/μl with the coinjection marker *myo-2p*::*mCherry*.

***myo-3p*::*pvf-1*** (FJ#133) cDNA rescue construct was made by excising *pvf-1* from FJ#128 and inserting it into FJ#16 (which contains *myo-3p* in pPD49.26) using NheI and KpnI. pFJ#133 was injected into *akIs201* at 10 ng/μl with *coel*::*GFP* at 25 ng/μl and pBluescript SK at 65 ng/μl to make *pzEx423*.

***myo-3p*::*pvf-1*::*gfp*** (FJ#150) was made by subcloning GFP in-frame at the C-terminus of PVF-1 using an introduced Not I site right before the stop codon in FJ#133. FJ#150 was injected at 50ng/μl with the co-injection marker *unc-122p*::*RFP* [90].

***hsp-16*.*2*::*pvf-1*** (FJ#151) heat-shock inducible construct was made by subcloning pvf-1 from FJ#133 into pPD95.77 (which contains the *hsp-16*.*2* promoter) using Nhe I and Kpn I. FJ#151 was injected at 10 ng/μl with *unc-122p∷GFP* at 25 ng/μl and pBluescript SK at 65 ng/μl to make *simEx7*.

### RNAi screening

RNA interference (RNAi) was performed using the FJ1300 strain optimized for enhanced neuronal RNAi [*lin-35 (n745); uIs69 (unc-119p*::*sid-1)*, *sid-1 (pk3321)*] [42] that also contained both channelrhodopsin-2 (ChR2) expressed in ASH sensory neurons [*lite-1 (ce314)*, *ljIs114 (gpa-13p*::FLPase, *sra-6p*::*FTP*::ChR2::YFP*)*] [38] and GLR-1::GFP expressed under control of the *glr-1* promoter [*glr-1p*::GLR-1::GFP *(nuIs25)*]. An RNAi sub-library targeting genes with cell adhesion molecule domains (gift from Joshua Kaplan, Massachusetts General Hospital [91]) was screened at least 3 times in triplicate using the optoASH assay (see below) on populations of worms in 24-well plates. Clones for which knockdown resulted in an impaired stimulated reversal response but wild type-like gross locomotor activity in the majority of wells for each experiment were further analyzed at the individual worm level. RNAi clones for this sub-library and for controls were selected from a genome-wide library consisting of *Escherichia coli* (*E*. *coli HT115 (DE3)*) containing RNAi constructs in the T7-tailed L4440 vector [92,93]. Bacteria were maintained in the presence of 50 μg/ml ampicillin (Sigma #A9518) and 15 μg/ml tetracycline (Sigma #87218) for plasmid selection. One generation RNAi was performed as follows: 1 ml overnight cultures of bacteria carrying RNAi plasmids were grown at 37°C in Luria broth containing 50 μg/ml ampicillin. After adding all-*trans* retinal (ATR, Sigma #R2500) dissolved in ethanol to a final concentration of 100 μM, 30 μl of these cultures were spotted on 24-well nematode growth medium agar plates containing 50 μg/ml carbenicillin (Sigma #C1389) and 5 mM isopropyl-β-D-thiogalactopyraniside (Sigma #PHG0010). Plates were allowed to dry uncovered for 1 h in a chemical hood and were then either used immediately or wrapped in aluminum foil to protect the light-sensitive ATR and stored at room temperature overnight. Two gravid adult FJ1300 worms were placed in each well of the spotted NGM plate for ~24 h after which the adults were removed, and the deposited eggs were allowed to develop to the 4^th^ larval stage (L4) before undergoing behavioral testing (optoASH assay described below).

### Behavioral assays

All behavioral assays were performed by an experimenter blinded to the genotypes of the animals being tested. *Nose-touch* assays were performed as described previously [27]. Briefly, worms were placed on 60 mm NGM agar plates containing a thin lawn of OP50 *E*. *coli* spotted the day before the assay and then assayed for responsiveness to light touch on the nose as determined by perpendicular contact with a human eyelash during forward locomotion. Ten trials were performed for each animal, and backward movement greater than the distance from the nose to the terminal bulb of the pharynx was counted as a locomotor reversal. All statistical analyses were performed using Student’s *t* test (for two genotypes or conditions) or ANOVA followed by multiple comparison tests (for greater than two genotypes) as specified in the individual figure legends. Heat shock-inducible rescue experiments were performed as described previously [94]. Briefly, worms were raised at 20°C until the L4 stage and then heat-shocked at 30°C for 3 h. Animals were allowed to recover at 20°C for 16 h and assayed for nose-touch responses as adults. *Optogenetic activation of ASH-dependent nose touch response (*OptoASH) was performed similarly to what has been described previously [75]. Animals used for OptoASH assays were grown for one generation in the dark on NGM agar plates spotted with OP50 and 100 μM all-*trans*-retinal (ATR). For the OptoASH assay, worms were transferred to an NGM agar plate spotted with OP50 (without ATR) and illuminated with 1 s pulses of blue light (0.47 mW/mm^2^) from a mercury bulb filtered through a GFP excitation filter under 32x total magnification on a Leica MZ16F microscope. Locomotor reversals (backward movement greater than the distance from the nose to the terminal bulb of the pharynx) observed during or immediately after illumination was counted as a response. *Thrashing assays* were performed as described previously [47]. Briefly, individual worms were picked to a 5 μl droplet of M9 buffer (KH_2_PO_4_, 22.0 mM; Na_2_HPO_4_, 42.3 mM; NaCl, 85.6 mM autoclaved, plus 1 mM sterile MgSO_4_), allowed to acclimate for 1–2 minutes until thrashing began. The number of thrashes, as defined by movement of both the head and tail from one side of the midpoint of the body to the other and back, was counted over the course of 30 seconds. *Aldicarb-induced paralysis assays* were performed similarly to what has been described previously [95]. This assay monitors the rate of paralysis induced by the acetylcholinesterase inhibitor aldicarb. Treatment with aldicarb leads to a buildup of acetylcholine at the NMJ, excessive muscle activation and paralysis over time. Briefly, assays were performed on 35 mm NGM agar plates containing 1 mM aldicarb and spotted with OP50. Aldicarb plates were used one day after preparation. Twenty worms were transferred to each aldicarb plate and incubated for 30 minutes at 20°C. Worms were then tested for paralysis in 15 minute intervals as follows: a platinum wire worm pick was used to prod each worm on the nose twice, and the fraction of fully paralyzed worms was recorded. *Spontaneous locomotor reversals* were analyzed as previously described [96]. Briefly, individual worms were transferred to an unspotted NGM plate using halocarbon oil. After a two-minute acclimatization, the number of times forward-moving animals reversed directions (greater than the distance from the nose to the terminal bulb of the pharynx) was counted over a 5-minute period.

### Epifluorescence microscopy

All epifluorescence imaging was performed with a Carl Zeiss AxioImager M1 microscope with a 100x Plan Aprochromat objective (1.4 numerical aperture) with GFP and RFP filter cubes. Images were acquired with an Orca-ER charge-coupled device camera (Hamamatsu), using MetaMorph, version 7.1 software (Molecular Devices). All L4 animals were immobilized with 30 mg/ml 2,3-butanedione monoxamine (Sigma # B0753) for 6–8 minutes before imaging. At least 20 animals were measured for each genotype across multiple experiments and statistical analyses were performed by Student’s *t* test (for two genotypes) or ANOVA followed by Tukey’s multiple comparisons test (for greater than two genotypes). Control genotypes were always assayed on the same day along with 0.5 μm FluoSphere yellow-green fluorescent beads (Thermo Fisher #F8803) to normalize for daily fluctuations in arc lamp intensity.

### Imaging of fluorescent puncta

To quantify fluorescent puncta in the ventral nerve cord (VNC), maximum intensity projections of 1 μm Z-series stacks were made. Images were acquired from a 60–100 μm section of the anterior VNC, posterior to the RIG and AVG neuronal cell bodies. To quantify mCherry puncta in the nerve ring (NR), maximum intensity projections of 20 μm Z-series stacks were made. Exposure settings and gain were optimized to fill the 12-bit dynamic range without saturation and set identically for all images acquired within an experiment with a given fluorescent marker. Line scans of VNC or NR puncta were made using MetaMorph (version 7.1) software. Line scan data was analyzed in an automated manner with Igor Pro (v6, Wavemetrics), using custom-written software as described previously [51]. Puncta were identified based on a minimum peak width of 0.3 μm and minimum peak threshold that was 4 standard deviations above the background fluorescence in the VNC. The same settings were used for analysis of all the data from the same fluorescent marker in wild-type and mutant backgrounds. Puncta intensities were normalized to the average fluorescent bead intensity for the corresponding day. Graphs of puncta intensities display maximum puncta intensity values normalized to wild-type controls. Puncta widths were calculated by measuring the width of each punctum at 50% of the maximal peak fluorescence intensity. Puncta densities were measured by calculating the average number of puncta per 10 μm along the VNC.

### Imaging of mCherry fluorescence in AVA soma

AVA soma were imaged with a Carl Zeiss Axioimager equipped with a 100X objective (NA 1.4) and Orca-ER charge-coupled device camera (Hamamatsu) using MetaMorph, version 7.1 software (Molecular Devices) as described above. Images were acquired at 25% transmittance, 100ms exposure settings and 1 by 1 binning. Fluorescence intensities were normalized to wild-type controls for each day of imaging. To quantify mCherry fluorescence in the soma of AVA neurons, maximum intensity projections of 5 μm Z-series stacks were made. Exposure settings and gain were optimized to fill the 12-bit dynamic range without saturation and set identically for all images. A region of interest (ROI) was drawn around the soma using ImageJ (NIH) and the ROI area and integrated density (the product of area and mean gray intensity) were used for quantification.

### DiI labeling of sensory neurons

Worms were rinsed from growth plates into a 1.5 ml microcentrifuge tube using 1 ml M9 buffer, spun at 400 x *g* for 2 minutes, and resuspended in 1 ml M9 to which 5 μ of 2 mg/ml DiI (1,1-Dioctadecyl-3,3,3’,3’-tetramethylindocarbocynanine perchlorate)(Sigma #468495) in dimethyl formate was added. Worms suspensions were incubated overnight on a nutating rocker. Worms were then washed twice with M9 and transferred onto 2% agarose pads containing 3.4 mM levamisole. Maximum intensity projections of 10 μm Z-series stacks were made from laterally-oriented worms, and ASH was identified by position relative to other amphid sensory neurons.

### Confocal imaging and analysis

#### Imaging of SEP::mCherry::GLR-1

Fluorescence imaging of SEP::mCherry::GLR-1 was performed using a Zeiss LSM800 confocal microscope with a 63X Plan Apochromat objective (N.A. 1.4). L4 animals were immobilized by incubation in a 5 μl droplet of M9 containing 2.3 mM levamisole for 5 minutes and transferred to a 2% agarose pad containing 3.4 mM levamisole (tetramisol, Sigma #T1512). Orthogonal projections of 7 μm Z-series stacks of ~80 μm lengths of the anterior VNC were made after applying a single pixel filter to reduce noise. Acquisition settings for all images (with the exception of *unc-11* mutant images, see below) were as follows. Acquisition area: 100 μm x 60 μm. 488 nm laser: 8.94%, 776 V gain. 561 nm laser: 5.19%, 867 V gain. For imaging sessions that included *unc-11* worms, the following settings were used. Acquisition area: 100 μm x 60 μm. 488 nm laser: 2.4%, 776 V gain. 561 nm laser: 2.8%, 867 V gain. To quantify SEP and mCherry fluorescence in VNC puncta, a separate average background threshold was calculated for mCherry and SEP images from a representative subset of WT VNC image sets using ImageJ (NIH). These thresholds were then applied to all respective mCherry or SEP images for analysis. The same ROI was then drawn around the VNC in both the SEP and mCherry images and the integrated density (product of area and mean gray intensity) of thresholded VNC puncta were measured. Following quantification, orthogonal projections were globally processed in Photoshop (Adobe) to further reduce noise and increase contrast equivalently for all channels.

#### Fluorescence recovery after photobleaching

A pre-bleach image was obtained with a 1 μm total depth Z-series using a 488 nm laser at 5.8% and a 561 nm laser at 3.6%. A 50% cropped portion of the sample (for a total of 16 μm in length and 8 μm from each edge of the image area) was then bleached using the 488 nm laser at 30% and the 561 nm laser at 10%. Post-bleach images were taken using the pre-bleach settings immediately following the bleach, and then 2.5 minutes, 5 minutes, and 10 minutes after bleaching. To quantify the fluorescence, thresholds were set separately for the 488nm and 561nm channels and applied to the respective 488 nm channel image and the 561 nm channel image for each time point. The ROI for FRAP quantification for each individual dataset was a 50% cropped portion of the bleached area (for a total of 8 μm in length and 4 μm from each edge of the bleached area) to avoid measuring fluorescence from receptors that diffused into the bleached area from the non-bleached area of the VNC. Total fluorescence in the ROI was recorded from the pre-bleach, post-bleach, 2.5 minutes, 5 minutes, and 10 minutes images and normalized to the fluorescence of the pre-bleach image to determine the percent fluorescence recovery after photobleaching. Control and mutant genotypes were always assayed on the same day.

### Statistical analysis

All Statistical analyses were performed using Prism 7 (GraphPad) software using Student’s *t* test or one-way or two-way ANOVA with posthoc tests as appropriate. Specific tests used are indicated in the Figure legends.

## Supporting information

S1 Fig Reversal behavior of mutants with defective glutamate signaling. (A) Simplified version of the locomotion reversal circuit after activation of ASH by mechanical nose touch or optogenetics (OptoASH). ASH releases glutamate that activates downstream command interneurons via *glr-1* AMPARs. (B) OptoASH assay of mutants with known glutamate signaling or GLR-1 trafficking defects mirrors their known response to classical nose touch stimulation. Mean ± SEM are shown (n ≥ 24 worms from ≥ 3 experiments). *p ≤ 0.05 vs WT, ANOVA followed by Tukey’s multiple comparisons test.
(PDF)

S2 Fig Data used in S1 Fig. (PDF)

S3 Fig Data used in Fig 1. (PDF)

S4 Fig Data used in Fig 2. (PDF)

S5 Fig Loss of VERs does not cause gross changes in GLR-1 neurites. (A) Representative images of GLR-1::GFP puncta (*pzIs12*) in the VNC of WT and *ver* mutants. (B,C) Quantification of the relative intensity, width, and density of GLR-1::GFP puncta in the VNC of WT and *ver* mutants. Mean ± SEM are shown. (n > 20 worms from 3 experiments). *p ≤ 0.05 vs WT, Student’s *t* test. (D) Representative images of GFP-tagged synaptobrevin (SNB-1::GFP)(*nuIs125*) in the VNC of GLR-1-expressing neurons of WT and *ver* mutants. (E) Quantification of the relative intensity, width, and density of SNB-1::GFP puncta in the VNC of WT and *ver* mutant worms. Mean ± SEM are shown (n ≥ 15 worms from 3 experiments). No significant differences (p > 0.05) were observed.
(PDF)

S6 Fig Data used in S5 Fig. (PDF)

S7 Fig Loss of VER-1 or VER-4 does not affect the total levels of somatic GLR-1 in AVA. (A) Schematic of SEP::mCherry::GLR-1 fluorescence in different subcellular compartments. mCherry is fluorescent regardless of its subcellular localization while SEP fluorescence is quenched in the acidic endosomal environment and is therefore detectable only when GLR-1 is at the cell surface. (B) Representative mCherry fluorescence images (total GLR-1) of SEP::mCherry::GLR-1 in the soma of AVA in WT and *ver* mutants. (C) Relative measurements of AVA soma area and total somatic mCherry fluorescence in *ver* mutant worms were unchanged from WT levels. Mean ± SEM are shown. (n ≥ 25 worms from 3 experiments). No significant differences (p > 0.05) were observed. ANOVA followed by Tukey’s multiple comparisons test.
(PDF)

S8 Fig Data used in S7 Fig. (PDF)

S9 Fig Data used in Fig 3. (PDF)

S10 Fig Data used in Fig 4. (PDF)

S11 Fig Data used in Fig 5. (PDF)

S1 Table Data used in Fig 1D. (XLSX)

S2 Table Data used in Fig 2E. (XLSX)

S3 Table Data used in Fig 2F. (XLSX)

S4 Table Data used in S5B Fig. (XLSX)

S5 Table Data used in S5C Fig. (XLSX)

S6 Table Data used in S5E Fig. (XLSX)

S7 Table Data used in Fig 4D. (XLSX)

S8 Table Data used in Fig 5H. (XLSX)
