# Supplementary figures and images for: VER/VEGF receptors regulate AMPA receptor surface levels and glutamatergic behavior
Source: PLoS Genet. 2021 Feb 9;17(2):e1009375. doi: 10.1371/journal.pgen.1009375 (PMC7899335; doi:10.1371/journal.pgen.1009375)

# S1 Fig

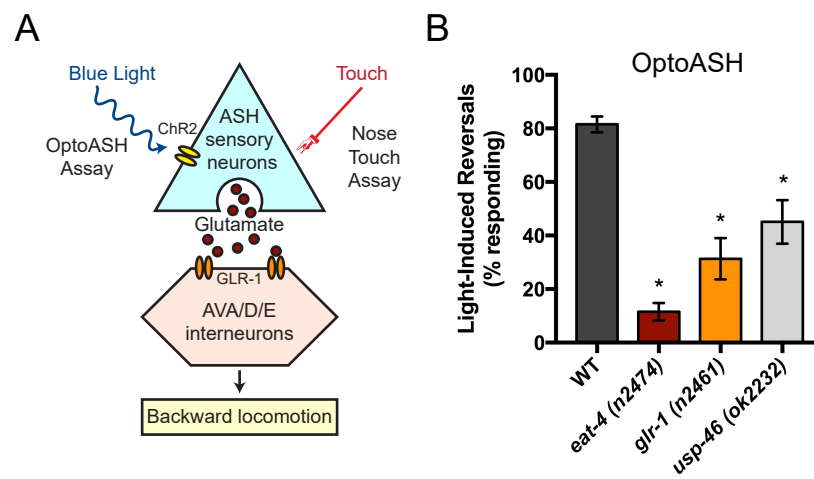

Supplement: S1 Fig — (A) Simplified version of the locomotion reversal circuit after activation of ASH by mechanical nose touch or optogenetics (OptoASH). ASH releases glutamate that activates downstream command interneurons via glr-1 AMPARs. (B) OptoASH assay of mutants with known glutamate signaling or GLR-1 trafficking defects mirrors their known response to classical nose touch stimulation. Mean ± SEM are shown (n ≥ 24 worms from ≥ 3 experiments). *p ≤ 0.05 vs WT, ANOVA followed by Tukey’s multiple comparisons test. (PDF) [file pgen.1009375.s001.pdf]

## S2 Fig

A

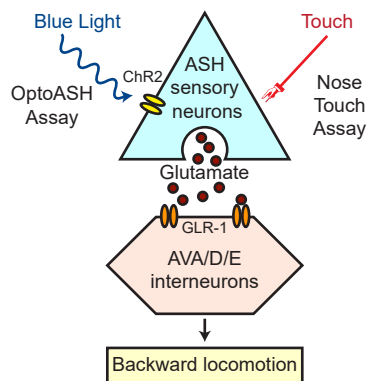

B

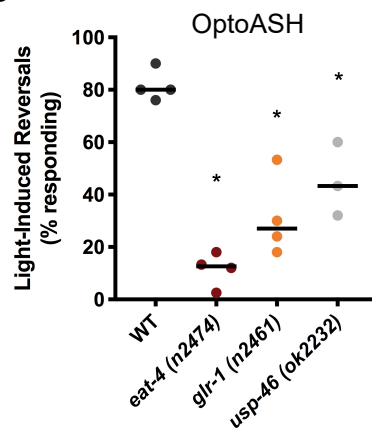

Supplement: S2 Fig — (PDF) [file pgen.1009375.s002.pdf]

S3 Fig

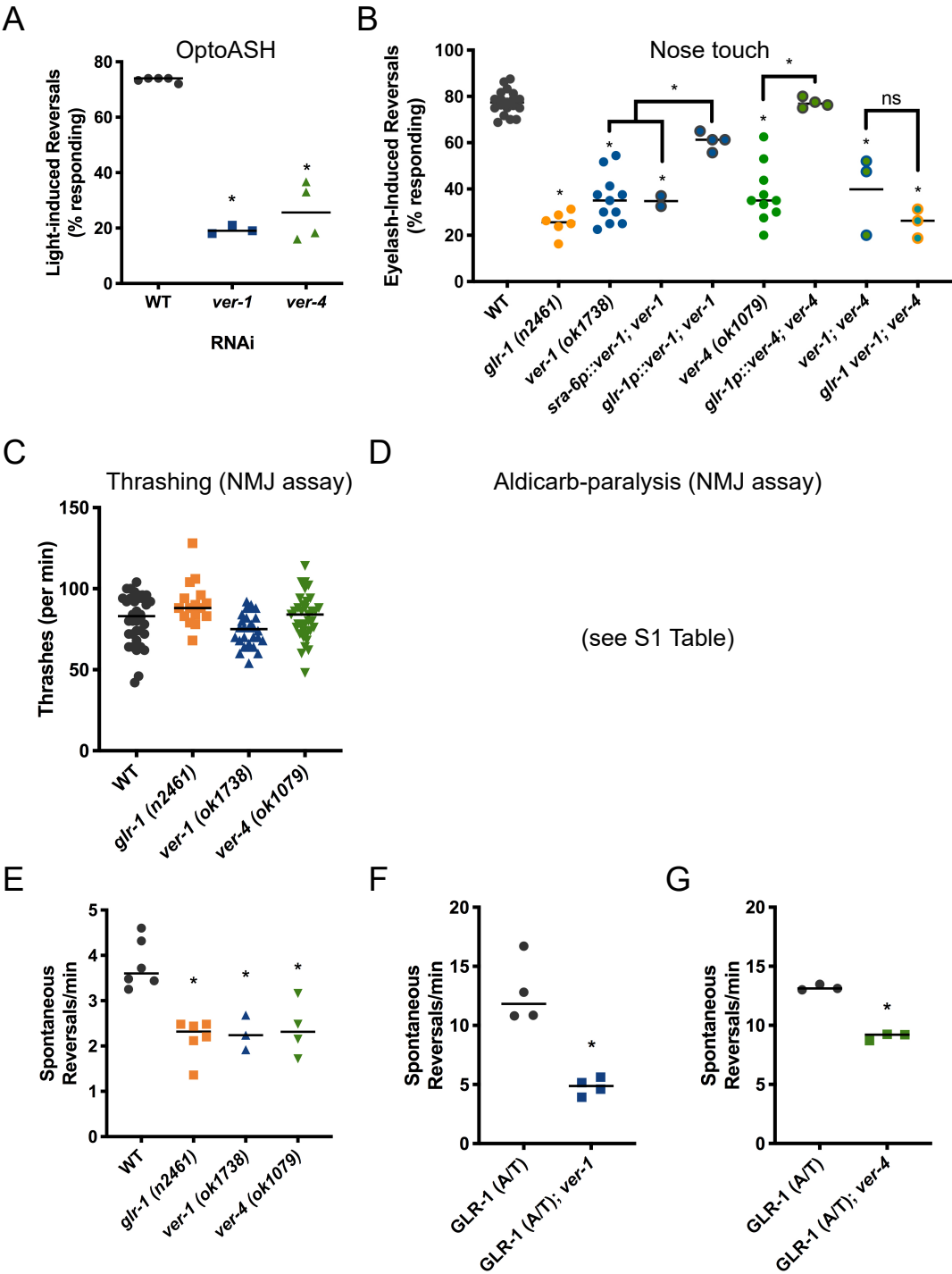

Supplement: S3 Fig — (PDF) [file pgen.1009375.s003.pdf]

S4 Fig

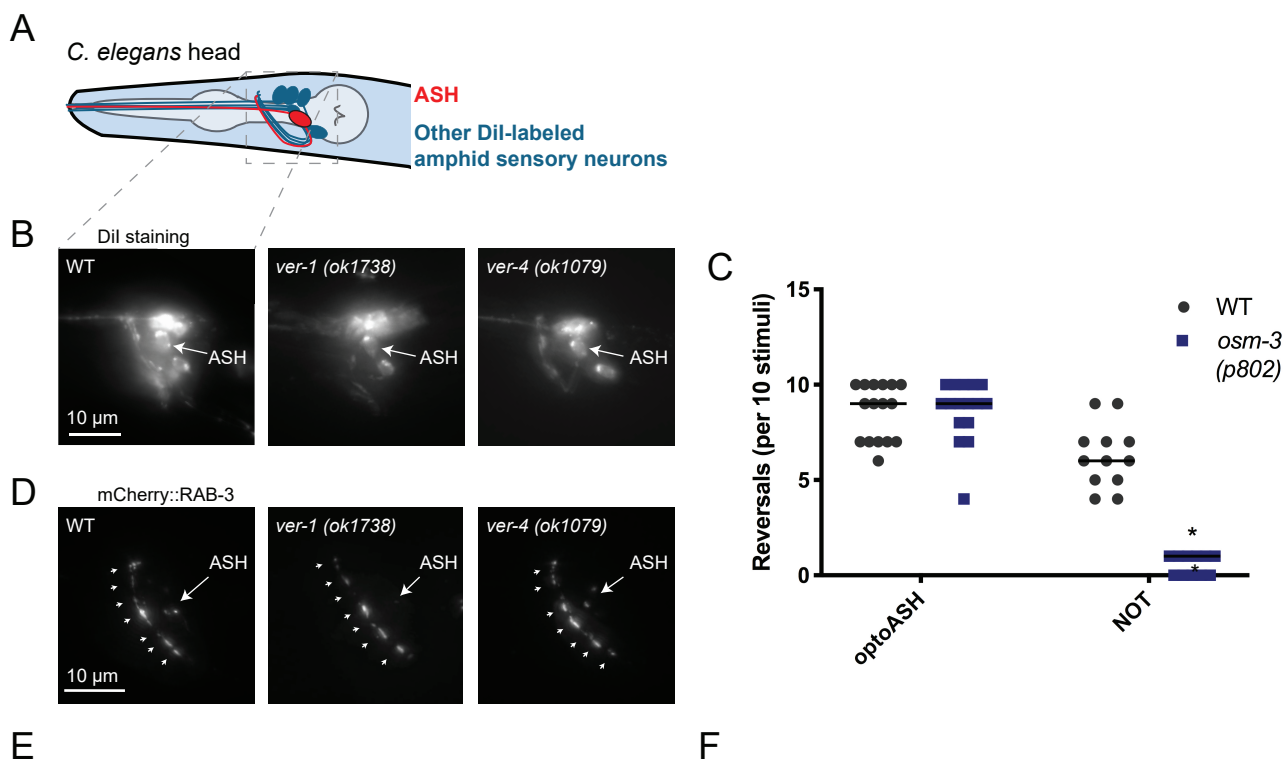

(see S2 Table)

(see S3 Table)

Supplement: S4 Fig — (PDF) [file pgen.1009375.s004.pdf]

S5 Fig

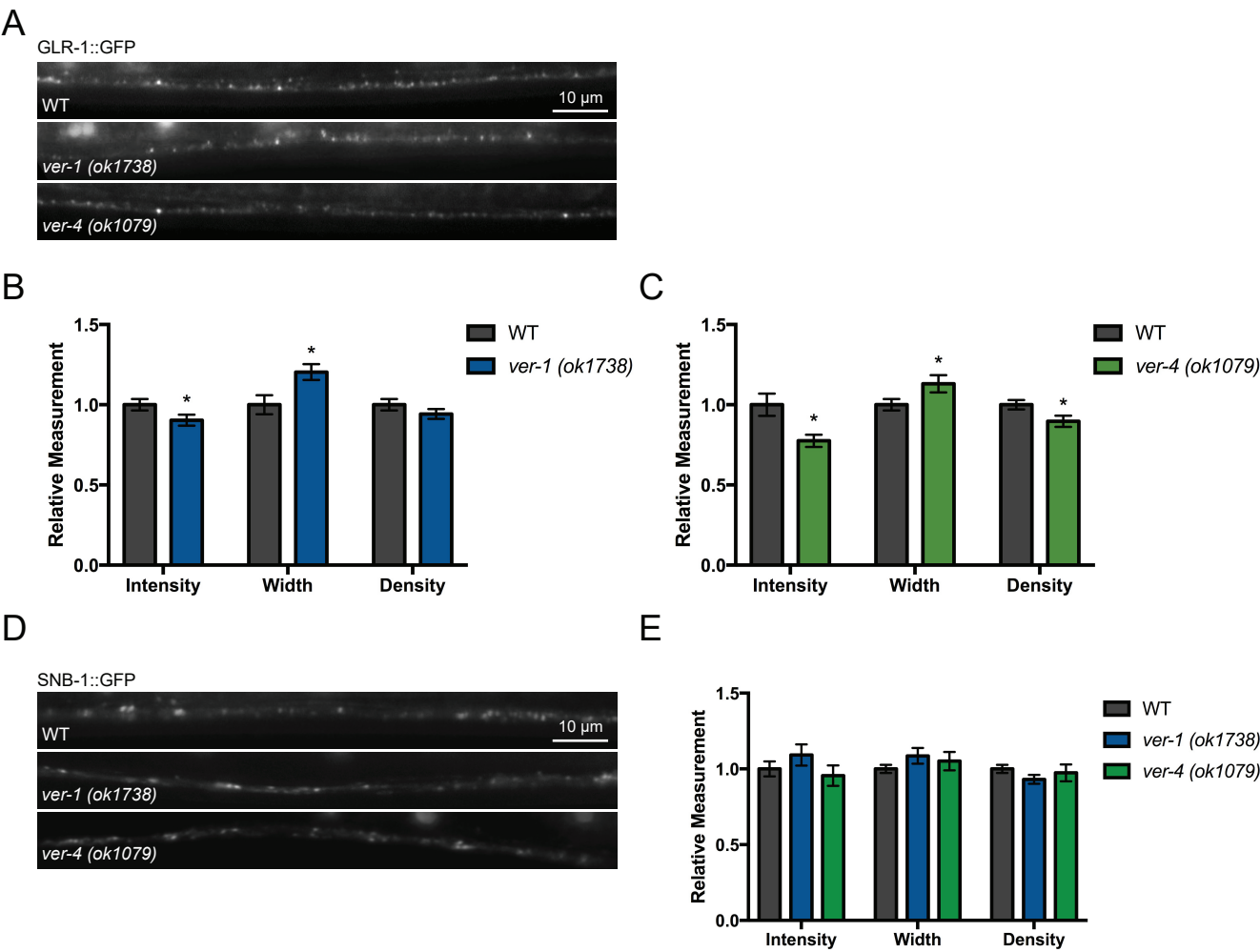

Supplement: S5 Fig — (A) Representative images of GLR-1::GFP puncta (pzIs12) in the VNC of WT and ver mutants. (B,C) Quantification of the relative intensity, width, and density of GLR-1::GFP puncta in the VNC of WT and ver mutants. Mean ± SEM are shown. (n > 20 worms from 3 experiments). *p ≤ 0.05 vs WT, Student’s t test. (D) Representative images of GFP-tagged synaptobrevin (SNB-1::GFP)(nuIs125) in the VNC of GLR-1-expressing neurons of WT and ver mutants. (E) Quantification of the relative intensity, width, and density of SNB-1::GFP puncta in the VNC of WT and ver mutant worms. Mean ± SEM are shown (n ≥ 15 worms from 3 experiments). No significant differences (p > 0.05) were observed. (PDF) [file pgen.1009375.s005.pdf]

S6 Fig

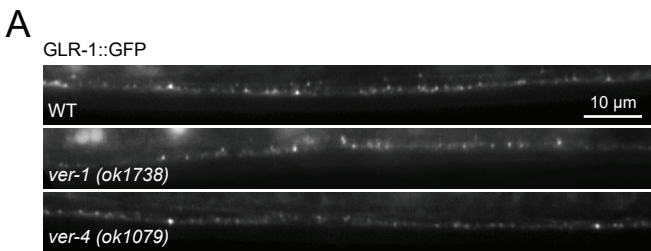

B

C

(see S4 Table)

(see S5 Table)

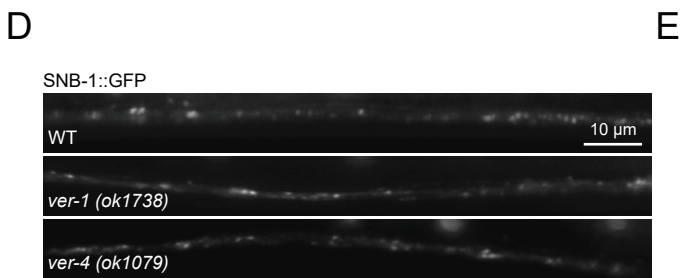

(see S6 Table)

Supplement: S6 Fig — (PDF) [file pgen.1009375.s006.pdf]

S7 Fig

A

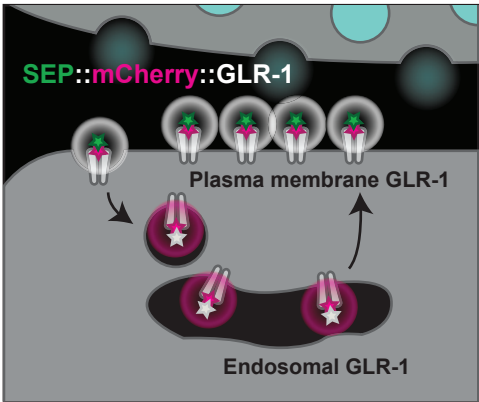

B

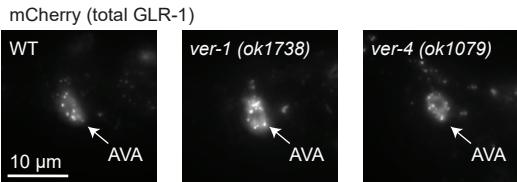

C

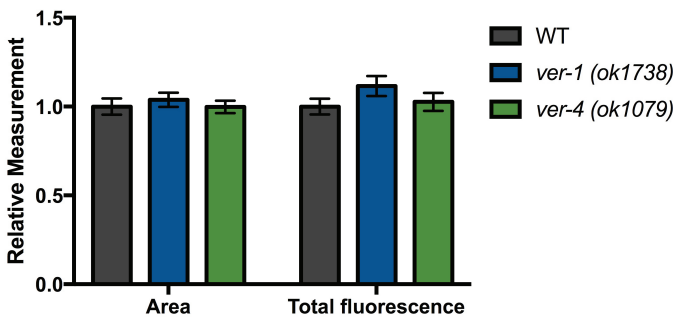

Supplement: S7 Fig — (A) Schematic of SEP::mCherry::GLR-1 fluorescence in different subcellular compartments. mCherry is fluorescent regardless of its subcellular localization while SEP fluorescence is quenched in the acidic endosomal environment and is therefore detectable only when GLR-1 is at the cell surface. (B) Representative mCherry fluorescence images (total GLR-1) of SEP::mCherry::GLR-1 in the soma of AVA in WT and ver mutants. (C) Relative measurements of AVA soma area and total somatic mCherry fluorescence in ver mutant worms were unchanged from WT levels. Mean ± SEM are shown. (n ≥ 25 worms from 3 experiments). No significant differences (p > 0.05) were observed. ANOVA followed by Tukey’s multiple comparisons test. (PDF) [file pgen.1009375.s007.pdf]

S8 Fig

A

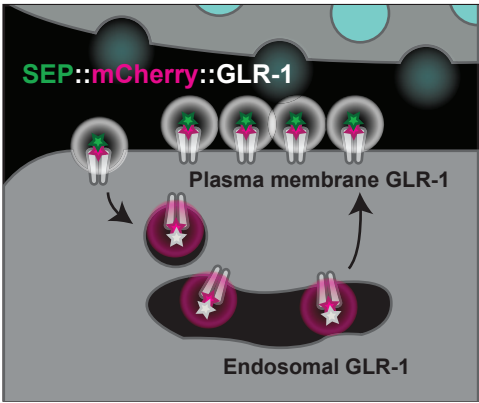

B

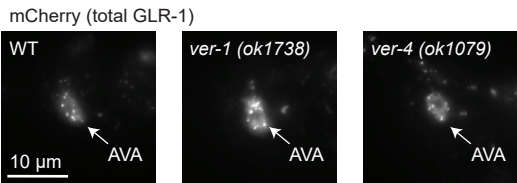

C

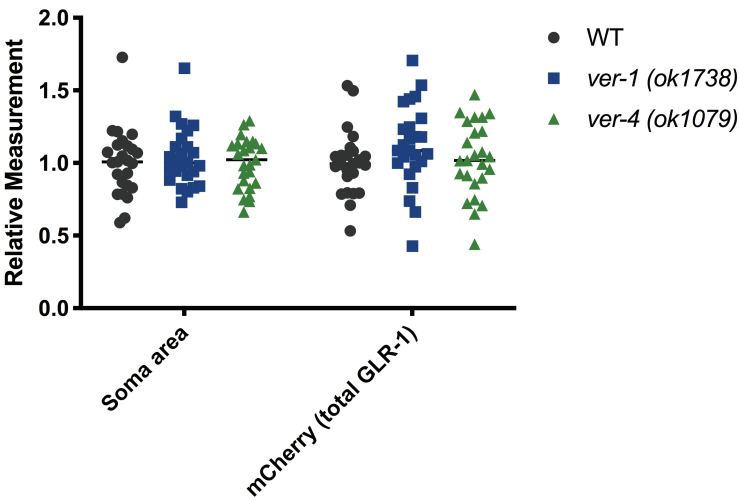

Supplement: S8 Fig — (PDF) [file pgen.1009375.s008.pdf]

# S9 Fig

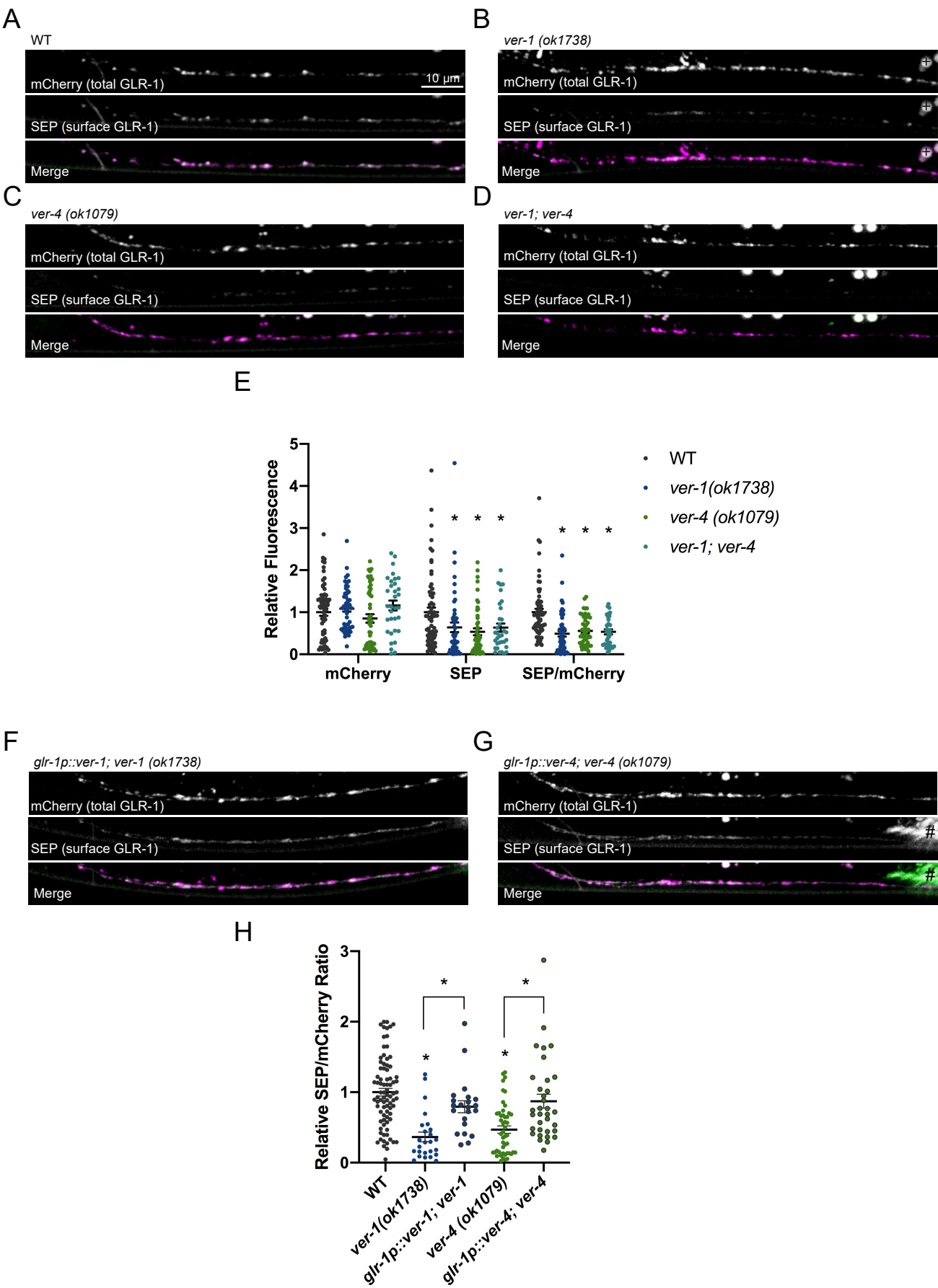

Supplement: S9 Fig — (PDF) [file pgen.1009375.s009.pdf]

S10 Fig

A

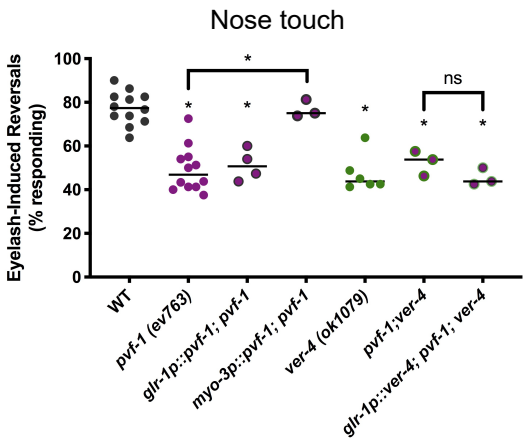

B

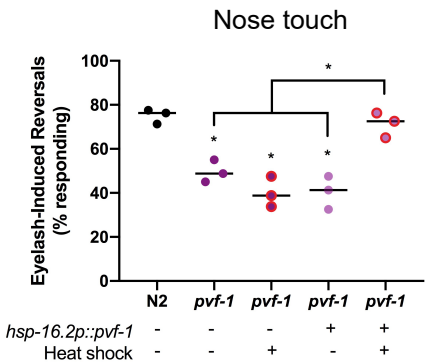

C

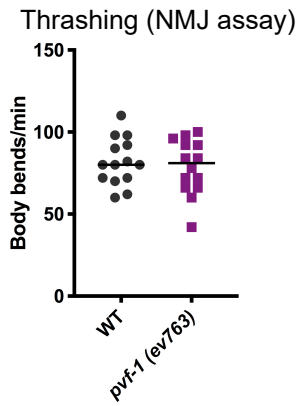

D

Aldicarb-paralysis (NMJ assay)

(see S7 Table)

E

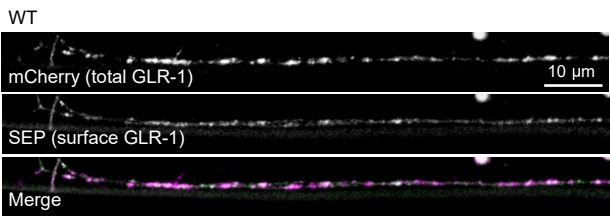

F

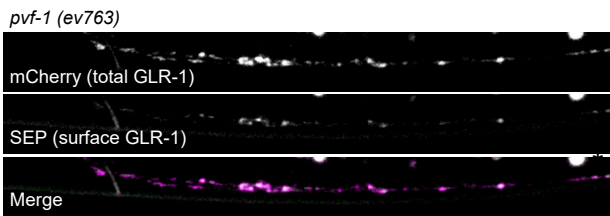

G

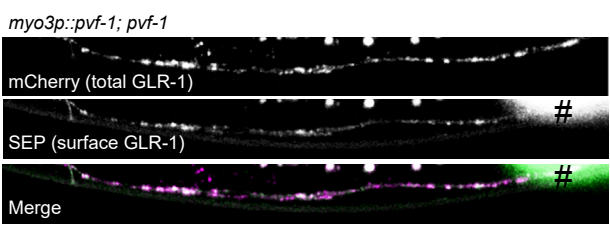

H

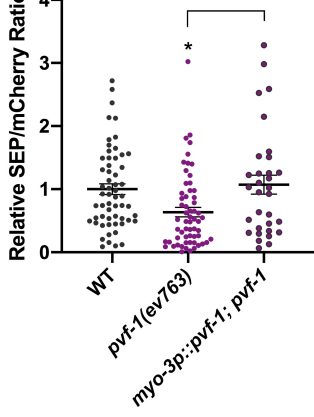

I

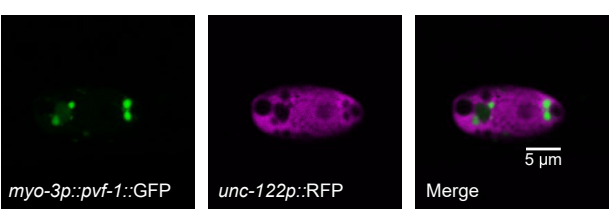

Supplement: S10 Fig — (PDF) [file pgen.1009375.s010.pdf]

S11 Fig

A

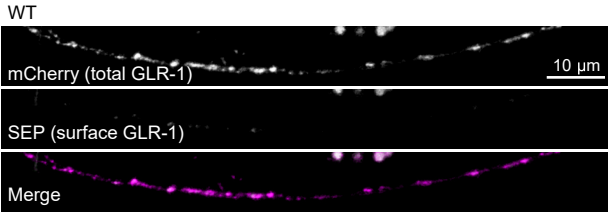

B

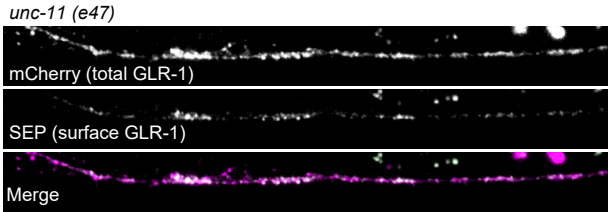

C

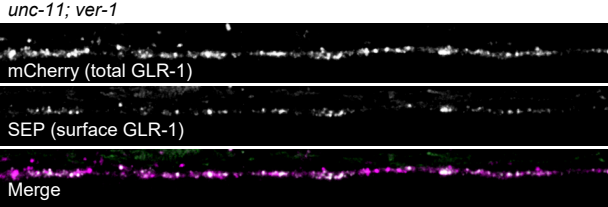

D

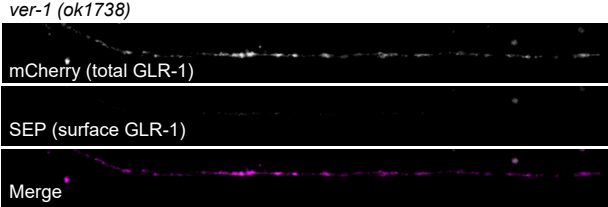

E

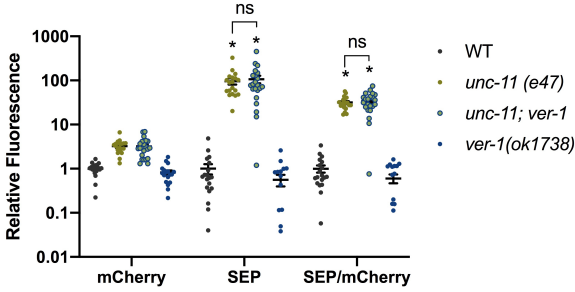

F

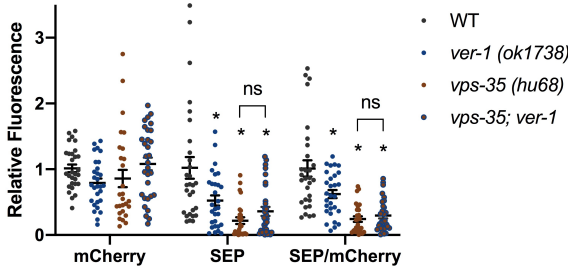

G

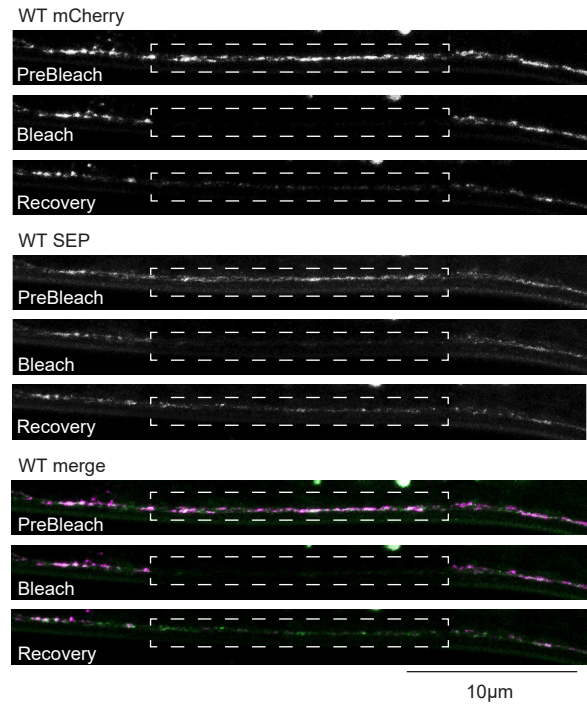

H

(see S8 Table)

Supplement: S11 Fig — (PDF) [file pgen.1009375.s011.pdf]
